# Antimicrobial, Antioxidant, and *α*-Glucosidase-Inhibitory Activities of Prenylated *p*-Hydroxybenzoic Acid Derivatives from *Oberonia ensiformis*

**DOI:** 10.3390/molecules30102132

**Published:** 2025-05-12

**Authors:** Lu-Lu Wang, Wei Tang, Zhuo Wang, Yi-Xiang Wang, Ning Li, Fu-Cai Ren

**Affiliations:** School of Pharmacy, Anhui Medical University, Hefei 230032, China; wanglulu2789@163.com (L.-L.W.); tangwei317822887@sina.com (W.T.); wangzhuo326@126.com (Z.W.); p965781684202503@163.com (Y.-X.W.)

**Keywords:** *Oberonia ensiformis*, prenylated aromatic compounds, antioxidant activity, *α*-glucosidase-inhibitory activity

## Abstract

Seven previously undescribed prenylated *p*-hydroxybenzoic acid derivatives, oberoniaensiformisins A–G, were isolated from an EtOH extract of the whole plant *Oberonia ensiformis*. Their structures were determined through spectroscopic analyses (IR, NMR) and HRESIMS analysis. The isolated compounds were tested for their antimicrobial, antioxidant, and *α*-glucosidase-inhibitory activity. Among them, compounds **6** and **12** exhibited potential antioxidant activity, while compounds **5**, **6**, **12**, **13**, and **15** showed varying degrees of *α*-glucosidase-inhibitory activity, with IC_50_ values ranging from 34.03 to 106.10 μg/mL.

## 1. Introduction

**Figure 1:**
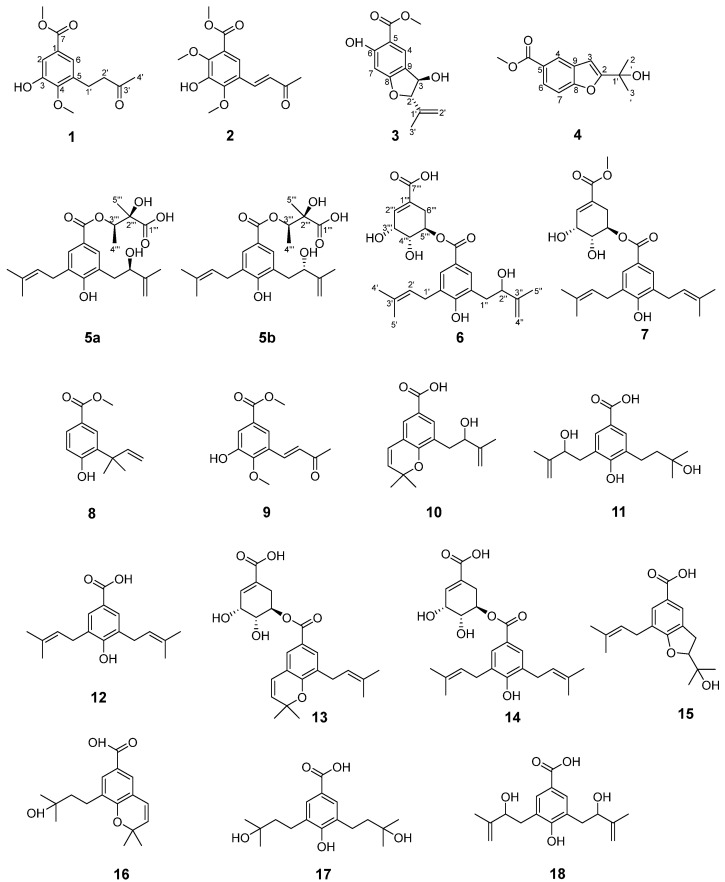
Structures of compounds **1**–**18**.

The Orchidaceae family represents the second-largest angiosperm family following Asteraceae, comprising approximately 28,000 species classified into 736 genera [1]. As a tribute to the 50th anniversary of the founding of New China in the 20th century, the monumental compendium of Chinese materia medica “Chinese Materia Medica “ documented as many as 155 medicinal orchid species belonging to 56 genera [2]. Beyond the orchid-based medicinal materials recorded in the *Pharmacopoeia of the People’s Republic of China*, an additional 47 species from 18 genera of Orchidaceae have been officially documented as source plants for traditional Chinese medicines and ethnic medicines in various regional medicinal material quality standards. Notably, many of these species are commonly used in local traditional and ethnic medicine practices, with *Oberonia ensiformis* being an example [3]. The whole herb of *O. myosurus Lindl.* has the effect of clearing heat and removing toxins, dispelling wind, and activating blood circulation. It is used by local ethnic minorities as a folk medicine mainly for the treatment of pneumonia, bronchitis, hepatitis, cystitis, and urinary tract infections and externally for the treatment of bruises, swelling and pain, and rheumatic bone pain [4]. In our previous study, we conducted a systematic investigation into the chemical constituents of *O. myosurus* Lindl [4]. To further explore and utilize the resources of plants within this genus, we carried out a detailed chemical study on the congeneric plant, *O. ensiformis*. As a result, 18 prenylated *p*-hydroxybenzoic acid derivatives, including 7 new compounds (**1**–**7**), were isolated and identified (Figure 1). All isolated compounds were evaluated for potential antimicrobial activity, antioxidant activity, and *α-*glucosidase-inhibitory activity, and the detailed results are presented in this paper.

## 2. Results

### 2.1. Structural Elucidation of the Isolated Compound

Compound **1** was isolated as a light-yellow oil. Its molecular formula, C_13_H_16_O_5_, was determined using positive-ion HRESI-MS, with an [M + Na]^+^ peak at *m*/*z* 275.0901 (calcd. for C_13_H_16_O_5_Na; 275.0895). The ^1^H NMR spectrum (Table 1) displayed two metacoupled aromatic doublets at δ_H_ 7.49 (d, *J* = 2.1 Hz, 1H) and 7.43 (d, *J* = 2.1 Hz, 1H), a methyl singlet at δ_H_ 2.17 (s, 3H), and two methoxy groups at δ_H_ 3.84 and 3.88 (each 3H, s). The ^13^C spectrum showed a total of 13 carbon resonances, including an aromatic ring [δ_C_ 126.7 (C-1), 115.2 (C-2), 148.9 (C-3), 149.2 (C-4), 134.1 (C-5), and 122.9 (C-6)], a carbonyl carbon δ_C_ 166.6, a methyl singlet δ_C_ 30.0, an ester methoxy group δ_C_ 52.3, and an aromatic methoxy group (δ_C_ 61.3). The NMR data of compound 1 were compared with those of methyl (E)-3-hydroxy-4-methoxy-5-(3-oxo-1,3-butadienyl) benzoate, a known compound from *O. myosurus* [4]. The key difference lies in the observation that both H-1′ and H-2′ in compound 1 exhibited methylene (CH_2_) signals rather than olefinic proton signals. Finally, compound 1 was identified as methyl 3-hydroxy-4-methoxy-5-(3-oxobutyl) benzoate and named as oberoniaensiformisin A.

**Figure 2:**
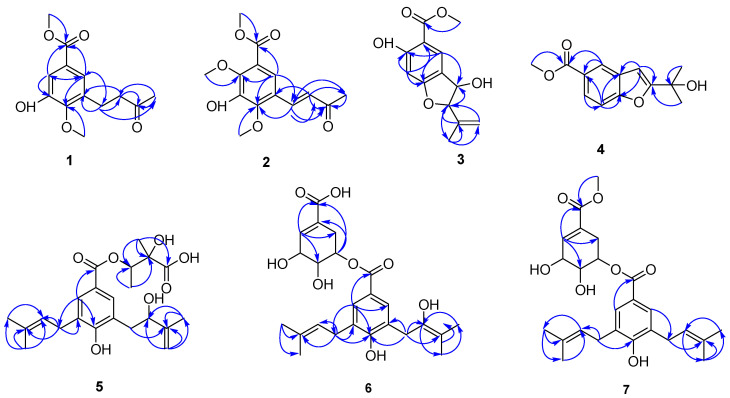
HMBC correlations of compounds **1**–**7**.

Compound **2** was isolated as a light-yellow oil. Its molecular formula was established as C_14_H_16_O_6_ based on positive-ion HRESIMS analysis, which revealed a [M + H]⁺ peak at *m*/*z* 281.1021 (calcd. for C_14_H_1_₇O_6_, 281.1025), corresponding to seven degrees of unsaturation. Comparative analysis of the NMR data between compounds **2** and **1** revealed the presence of an additional olefinic methylene proton pair and an aromatic methoxy signal in compound **2**, indicating that **2** is a structural analog of **1**. The ^1^H NMR spectrum of **2** exhibited a pair of vinyl protons at *δ*_H_ 7.73 (d, *J* = 16.4 Hz, 1H) and 6.77 (d, *J* = 16.4 Hz, 1H), a methyl singlet at *δ*_H_ 2.39 (s, 3H), and three methoxy singlets at *δ*_H_ 3.93, 3.95, and 4.00 (s, each 3H). The ^13^C NMR spectrum revealed signals corresponding to a benzene ring, two aromatic methoxy groups (*δ*_C_ 62.7 and 61.5), and an isoprene chain featuring a double bond (*δ*_C_ 128.5 and 137.1). These NMR data, combined with key HMBC correlations—such as those from the methoxy group at *δ*_H_ 3.95 (s, 3H) to C-2, from *δ*_H_ 4.00 (s, 3H) to C-4, from H-2′ to C-1′, C-3′, C-4′, C-4, and C-5, and from H-6 to C-1, C-3, and C-7—collectively indicated that compound **2** is a 2,3,4,5-trisubstituted benzoic acid methyl ester derivative. The structure features a hydroxy group and two methoxy groups positioned at C-3, C-2, and C-4, respectively. The side-chain structure was confirmed by the HMBC (Figure 2) correlations from H-4′ (*δ*_H_ 2.39) to C-1′ (*δ*_C_ 137.1), C-2′ (*δ*_C_ 128.5), and C-3′ (*δ*_C_ 198.6); from H-2′ (*δ*_H_ 6.77) to C-1′ (*δ*_C_ 137.1), C-3′ (*δ*_C_ 198.6), C-4′ (*δ*_C_ 27.7), C-4 (*δ*_C_ 150.0), and C-5 (*δ*_C_ 123.7); and from H-1′ (*δ*_H_ 7.73) to C-4 (*δ*_C_ 150.0), C-6 (*δ*_C_ 121.5), C-2′ (*δ*_C_ 128.5), and C-3′ (*δ*_C_ 198.6). The diagnostic coupling constant (*J* = 16.4 Hz) between H-1′ and H-2′ indicated that the double bond between C-1′ and C-2′ adopts an *E*-configuration. Therefore, the structure of compound **2** was determined to be methyl (*E*)-3-hydroxy-2,4-dimethoxy-5-(3-oxobut-1-en-1-yl) benzoate, and it was assigned the trivial name oberoniaensiformisin B.

**Figure 3:**
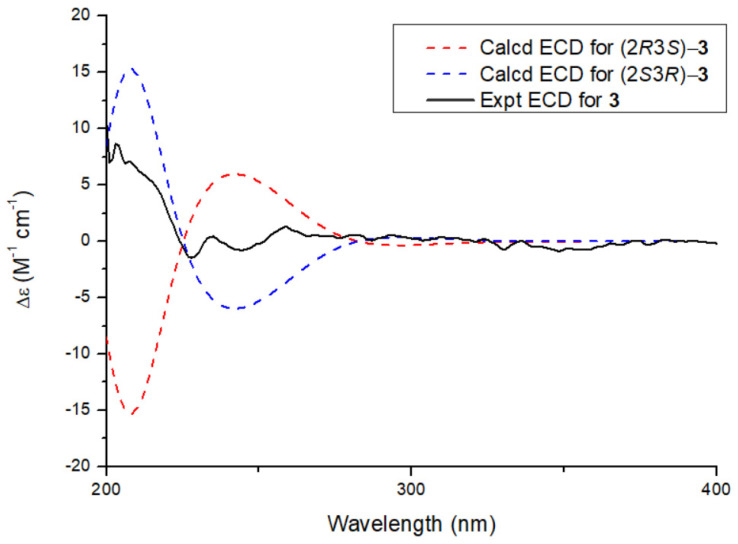
Experimental and calculated ECD spectra of compound **3**.

Compound **3** was obtained as a colorless oil. Its molecular formula, C_13_H_14_O_5_, was established by HR-ESI-MS analysis, which showed an [M + H]⁺ peak at *m*/*z* 251.0892 (calcd. for C_13_H_15_O_5_, 251.0919). The ^1^H NMR spectrum of **3** revealed signals characteristic of a 1,2,4,5-tetrasubstituted aromatic benzene ring, including two broad singlets at *δ*_H_ 7.30 (br s, H-7, 1H) and 7.01 (br s, H-4, 1H). Additionally, a methoxy group was observed at *δ*_H_ 3.95 (s, -OCH_3_, 3H). The spectrum also displayed signals for a terminal olefinic group at *δ*_H_ 4.94 and 5.10 (br s, each 1H) and a methyl group at *δ*_H_ 1.75 (s, 3H), indicating the presence of an isopropylene moiety (Table 2). The ^13^C NMR spectrum exhibited 13 carbon signals, including 8 characteristic benzofuran signals attributed to six aromatic carbons at *δ*_C_ 114.3 (C-4), 136.6 (C-5), 157.0 (C-6), 109.5 (C-7), 152.3 (C-8), and 113.3 (C-9) in the low-field region; one oxygenated carbon at *δ*_C_ 92.7 (C-2); one methine at *δ*_C_ 76.7 (C-3); and a carbonyl carbon (*δ*_C_ 170.3). Analysis of the NMR data of **3** revealed that compound **3** shares significant structural similarities with compound **4**, and compound **3** lacks a hydroxyl group at the C-6 position compared to compound methyl (2*R*,3*S*)-2,3-dihydro-3-hydroxy-2-(1-methylethenyl)-5-benzofurancarboxylate [5]. The presence of a hydroxyl group at the C-3 position was confirmed by HMBC correlations, from H-3 to C-2 and C-9, as well as from H-2 to C-3, C-8, C-9, and C-12. These correlations further indicated that the isopropene moiety was attached at the C-2 position. Based on the combined analysis of these data, the planar structure of the compound **3** was determined to be methyl 3,6-dihydroxy-2-(prop-1-en-2-yl)-2,3-dihydrobenzofuran-5-carboxylate. The relative configuration of compound **3** was assigned as 2S*3R*, supported by remarkable consistency in NMR chemical shifts with graphostrin D [5,6] and a computational analysis revealing a characteristic H-2-C-2-C-3-H-3 dihedral angle of 110° (Supplementary Materials). In addition, through DP4+ analysis of the carbon NMR data, the relative configuration of compound **3** was also determined to be 2S*3R* (Supplementary Materials). Further comparison between the experimental and calculated ECD spectra confirmed its absolute configuration as 2*S*3*R* (Figure 3). Thus, the structure of compound **3** was determined as shown in Figure 1 and was assigned the trivial name oberoniaensiformisin C.

Compound **4** was obtained as a colorless oil. The ^1^H NMR spectrum displayed signals corresponding to two methyl groups [*δ*_H_ 1.65 (s, H-2′, H-3′, 6H)], a methoxy group [*δ*_H_ 3.94 (s, -OCH_3_, 3H)], and an ABX-type benzene ring system [*δ*_H_ 8.28 (d, *J* = 1.8 Hz, H-4, 1H), 7.98 (dd, *J* = 8.6, 1.8 Hz, H-6, 1H), 7.54 (d, *J* = 8.6 Hz, H-7, 1H)]. Additionally, an alkene hydrogen signal was observed at *δ*_H_ 6.77 (s, H-3, 1H). The ^13^C NMR and HSQC spectra revealed 14 carbon signals, including a carbonyl carbon [*δ*_C_ 168.8 (C=O)], six aromatic carbons [*δ*_C_ 158.8(C-8), 130.1(C-5), 126.7(C-6), 126.2(C-9), 124.4(C-4), 111.9(C-7)], two alkene carbons [*δ*_C_ 167.0(C-2), 101.8(C-3)], a quaternary carbon signal [*δ*_C_69.7 (C-1′)], a methoxy carbon signal [*δ*_C_ 52.6 (-OCH_3_)], and two methyl carbon signals [*δ*_C_ 28.9 (C-2′, 3′)]. The key HMBC correlations from H-3 to C-2/C-5/C-8/C-9, along with those from H-2′ to C-2/C-1′/C-3′, unequivocally confirmed the attachment of the methyl group at the C-1′ position. This compound differs from compound **10** exclusively in possessing a methyl acetate substituent at C-5 [7]. Hence, compound **4** was assigned as methyl 2-(2-hydroxypropan-2-yl) benzofuran-5-carboxylatev and named as oberoniaensiformisin D.

Compound **5** was isolated as an apparent single component after repeated HPLC purification. Its molecular formula (C_22_H_30_O₇) was established by HRESIMS and NMR data. Additionally, the ^1^H and ^13^C NMR spectra indicated that compound **5** exists as a mixture of two prenylated *p*-hydroxybenzoic acid derivatives in a 1:1 ratio, suggesting the presence of two stereoisomers in solution. Structural analysis was subsequently conducted on one of the configurations, designated as **5a**. The ^1^H NMR spectrum (Table 3) displayed characteristic signals corresponding to one set of 1,3,4,5-tetrasubstituted benzene rings at *δ*_H_ 7.58 (s, H-2) and 7.58 (s, H-6). Additionally, signals corresponding to five methyl groups were observed at *δ*_H_ 1.73 (s, H-5″, 3H), 1.70 (s, H-4′, 3H), 1.69 (s, H-5′, 3H), 1.27 (s, H-4‴, 3H), and 1.22 (s, H-5‴, 3H). Three methylene groups were also identified: *δ*_H_ 3.26 (d, *J* = 6.9 Hz, H-1′, 2H), 2.80 (m, H-1″, 2H), and 4.90, 4.75 (d, *J* = 6.2 Hz, H-4″, 2H). A carbonyl resonance was detected at *δ*_C_ 165.1, along with a prenyl group characterized by the following signals: *δ*_H_ 3.26 (d, *J* = 6.9 Hz, 2H), 5.25 (t, *J* = 7.4 Hz, 1H), 1.70 (s, 3H), 1.68 (s, 3H), and *δ*_C_ 28.2, 122.3, 131.0, 25.5, and 17.6. The remaining signals at *δ*_H_ 2.81 (d, H-1″, 2H), 4.23 (d, *J* = 18.8 Hz, H-2″, 1H), 4.75, 4.90 (d, *J* = 6.2,3.7 Hz H-4″, each 1H), and 1.73 (3H, s, H-5″) and *δ*_C_ 37.7, 74.9, 147.2, 110.4, and 18.1 were assigned as a 2-hydroxy-3-methylbut-3-en-1-yl unit, which was confirmed by the HMBC correlations (Figure 2) from H-1″ to C-2″ and C-3″ and from H-5″ to C-2″, C-3″, and C-4″. The esterification group was established as a 2,3-dihydroxy-2-methylbutyryloxy unit based on HMBC correlations from H-4‴ (*δ*_H_ 1.22, d, *J* = 6.3 Hz) to C-3‴ (*δ*_C_ 74.2) and C-2‴ (*δ*_C_ 75.3) and from H-5‴ (*δ*_H_ 1.26) to C-1‴ (*δ*_C_ 175.8), C-2‴, and C-3‴. The chemical shift in H-3‴ (*δ*_H_ 5.09) indicated that the hydroxy group at C-3‴ was esterified, which was further confirmed by the HMBC correlation from H-3‴ to C-7 (*δ*_C_ 165.1).

Attempts to separate this mixture were consistently unsuccessful. Based on the HPLC chromatogram, the mixture exists in an approximate 1:1 ratio of the *R*-epimer and its stereoisomer [8,9,10].

Compound **6** was obtained as a colorless oil. Its molecular formula, C_24_H_30_O_8_, was established by positive-ion HRESIMS, which displayed a [M + H]⁺ peak at *m*/*z* 447.2022 (calcd. for C_24_H_31_O_8_, 447.2019), corresponding to ten degrees of unsaturation. The ^1^H and ^13^C NMR spectra exhibited 24 carbon signals, including those characteristic of a tetrasubstituted benzene ring: *δ*_C_ 120.3, 129.1, 128.2, 158.1, 126.1, and 130.7, along with corresponding proton signals at *δ*_H_ 7.49 (s, 1H) and 7.54 (s, 1H). The esterification group was established as a (4-hydroxy-3-(2-hydroxy-3-methyl-3-butenyl)-5-(3-methyl-2-butenyl)benzoic acid unit according to the HMBC correlations (Figure 2) from H-2 (*δ*_H_ 7.49, s) to C-1″ (*δ*_C_ 28.0) and C-6 (*δ*_C_ 130.7); from H-1′ (*δ*_H_ 3.25, d) to C-4 (*δ*_C_ 158.1), C-2′ (*δ*_C_ 122.1), C-4′ (*δ*_C_ 25.4), and C-3′ (*δ*_C_ 132.2); and from H-1″ (*δ*_H_ 2.75, d) to C-2″(*δ*_C_ 74.6), C-3″ (*δ*_C_ 147.1), and C-5 (*δ*_C_ 126.1) [11]. The remaining signals [*δ*_H_ 2.21, br dd, (*J* = 18.3, 4.4 Hz), 2.62, br. dd, (*J* = 18.3, 4.4 Hz), *δ*_C_ 27.4; *δ*_H_ 3.79, (s), *δ*_C_ 67.5; *δ*_H_ 5.15, dt, (*J* = 5.9); *δ*_C_ 70.2; *δ*_H_ 4.27 (br s), *δ*_C_ 65.5, *δ*_H_ 6.68 (br s); *δ*_C_ 138.8] could be recognized as a shikimic acid ester [12]. The esterification process involving the C-5‴ hydroxy group and the C-7 carboxyl of (4-hydroxy-3-(2-hydroxy-3-methyl-3-butenyl)-5-(3-methyl-2-butenyl)benzoic acid was substantiated through the chemical shift observed at H-5‴ (*δ*_H_ 5.15) and the HMBC correlation from H-5‴ to C-7. Consequently, the structure of compound **6** was elucidated and designated as oberoniaensiformisin F.

Similar to compound **5**, compound **6** was also identified as a mixture. Repeated attempts to separate this mixture proved unsuccessful. HPLC chromatographic analysis demonstrated an approximate 3:1 ratio between the target compound and its co-existing mixture components.

Compound **7** had a molecular formula of C_25_H_32_O_7_, as determined by its HRESIMS, ^1^H-NMR, and ^13^C-NMR data. A comprehensive analysis of the 1D and 2D NMR data (Table 4) revealed the presence of a nervogenic acid moiety. Furthermore, the NMR data of compound **7** indicated that its structure closely resembles that of oberoniamyosurusin K, with the key difference being the replacement of the shikimic acid ester in oberoniamyosurusin K with a methyl shikimate moiety [4]. Hence, the structure of compound **7** was established and named as oberoniaensiformisin G.

The known compounds (**8**–**18**) were identified by comparing their experimental NMR spectral data with the corresponding data reported in the literature. These compounds were characterized as methyl 4-hydroxy-3-(2-methylbut-3-en-2-yl)benzoate (**8**) [13], (*E*)-3-hydroxy-4-methoxy-5-(3-oxo-1,3-butadienyl)benzoic acid methyl ester (**9**) [4], liparacid A (**10**) [14], oberoniamyosurusin E (**11**) [4], nervogenic acid (**12**) [15], oberoniamyosurusin L (**13**) [4], oberoniamyosurusin K (**14**) [4], oberoniamyosurusins I (**15**) [4], 1iparacids C (**16**) [14], oberoniamyosurusin F (**17**) [3], and liparacid B (**18**) [14].

### 2.2. In Vitro Antioxidant, Antibacterial, and α-Glucosidase Activity of Compounds **1**–**18**

The antibacterial and antioxidant activities of all compounds (compounds **5** and **6** were performed using their epimeric mixtures) were evaluated using the 96-well plate microbroth dilution method and DPPH radical scavenging assay, respectively. However, none of the compounds exhibited bactericidal activity against the three tested bacterial strains: *Escherichia coli* ATCC 25922, *Staphylococcus aureus* subsp. *Aureus* ATCC 29213, and *Pseudomonas aeruginosa* ATCC 27853.

**Figure 4:**
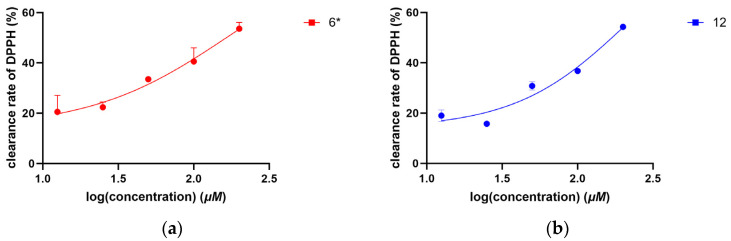
(**a**,**b**) Clearance rate of DPPH by compounds in vitro. Values are expressed as means ± SD. The asterisk (*) denotes a novel compound.

In the antioxidant assay, sodium L-ascorbate (positive control) demonstrated potent activity with an IC_50_ of 2.37 ± 0.11 μM. Among the tested compounds, only **6** and **12** exhibited weak DPPH radical scavenging activity (>50%), with IC_50_ values of 173.76 ± 23.92 μM and 185.36 ± 1.96 μM, respectively (Table 5, Figure 4).

**Figure 5:**
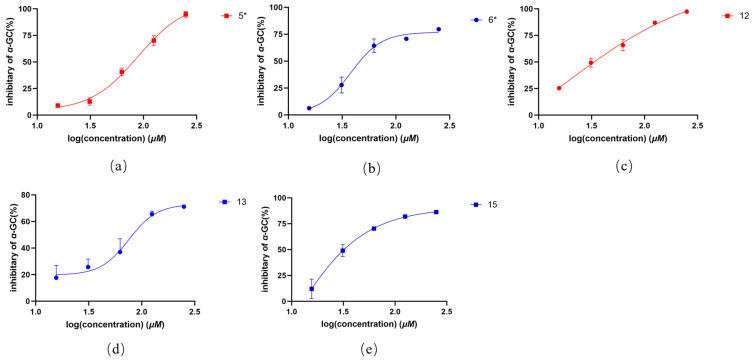
(**a**–**e**) Inhibition of *α*-glucosidase by compounds in vitro. Values are expressed as means ± SD. The asterisk (*) denotes a novel compound.

All isolated compounds were screened for *α*-glucosidase-inhibitory activity using the PNPG method, with acarbose as the positive control (IC_50_ = 0.66 ± 0.36 μg/mL). Among them, compounds **5**, **6**, **12**, **13**, and **15** showed moderate inhibitory effects, with IC₅₀ values ranging from 34.03 to 106.10 μg/mL (Table 6, Figure 5).

## 3. Discussion

The experiment was conducted to evaluate the antibacterial activity of three bacterial strains using the microbroth two-fold dilution method. The findings revealed that none of the tested compounds exhibited significant antibacterial activity. In the DPPH antioxidant assay, compounds **6** and **12** demonstrated a scavenging effect on DPPH radicals. In the *α*-glucosidase inhibition assay, compounds **5**, **6**, **12**, **13**, and **15** displayed varying degrees of inhibitory activity against α-glucosidase. Specifically, compounds **12** and **15** exhibited strong inhibitory effects, compounds **6** and **13** showed moderate activity, and compound **5** displayed weak activity. Preliminary structure–activity relationship (SAR) analysis suggests that the presence of isopentenyl chains at the C-3 and C-5 positions of the aromatic rings is a critical factor for α-glucosidase inhibition in isopentenylated benzoic acid derivatives. This conclusion is supported by the observation that compounds with isopentenyl chains at these positions tend to exhibit lower IC_50_ values, indicating higher inhibitory potency. Notably, although other derivatives (e.g., compounds **1**–**4**, **7**–**9**) also possess isopentenyl chains at either the 3 or 5 position, they were inactive. This suggests that carbonyl O-methylation at position 7 may negatively impact their activity [16,17].

Additionally, compounds **10**, **11**, **16**, **17,** and **18** also showed no significant activity against the enzyme. We hypothesize that this could be attributed to the specific positioning of the isopentenyl chain substitution and the presence of substituent groups on the chain, which may adversely affect the inhibitory activity. Further studies are warranted to explore additional in vitro activities of these isolated compounds and to validate the proposed SAR hypotheses.

## 4. Materials and Methods

### 4.1. General Experimental Procedures

NMR spectra were carried out on Bruker Avance III 600 spectrometers (Bruker BioSpin GmbH, Rheinstetten, Germany) with deuterated solvent signals used as internal standards, and chemical shifts (*δ*) are expressed in ppm. Electrospray ionization mass spectrometry (ESIMS) and high-resolution (HR)-ESIMS analyses were performed using an Agilent G6230 time-of-flight mass spectrometer (Agilent Technologies, Santa Clara, CA, USA). The infrared (IR) spectra were recorded using a Nicolet FTIR-iS20 spectrometer (Thermo Fisher Scientific, Waltham, MA, USA). Circular dichroism spectra were obtained using a Chirascan spectrometer (Applied Photophysics Ltd., Leatherhead, UK). Optical rotation was measured using a high-sensitivity polarimeter, MCP-150 (Anton Paar, Graz, Austria). Column chromatography (CC) was performed using silica gel (100–200 mesh and 300–400 mesh, Shanghai Haohong Scientific Co., Ltd., Shanghai, China), octadecylsilyl silica gel (12 nm, S-50 μm, YMC, Komatsu, Japan) and Sephadex LH-20 (25–100 µm, Cytiva, Uppsala, Sweden), and MCI-gel (75–150 µm, Mitsubishi Chemical Corporation, Tokyo, Japan). Thin-layer chromatography (TLC) was performed using silica gel GF254-precoated plates (Shanghai Haohong Scientific Co., Ltd., Shanghai, China), and spots were visualized by heating silica gel plates sprayed with 10% H_2_SO_4_ in EtOH.

### 4.2. Plant Material

The whole *O. myosurus* plants were collected in October 2021 from the Xishuangbanna Dai and Yi Autonomous Prefecture, Yunnan Province, People’s Republic of China. The plant was identified by Dr. Fucai Ren of Anhui Medical University. A voucher specimen (AHMU-R02) was deposited at the School of Pharmacy of Anhui Medical University, China.

### 4.3. Extraction and Isolation

**Figure 6:**
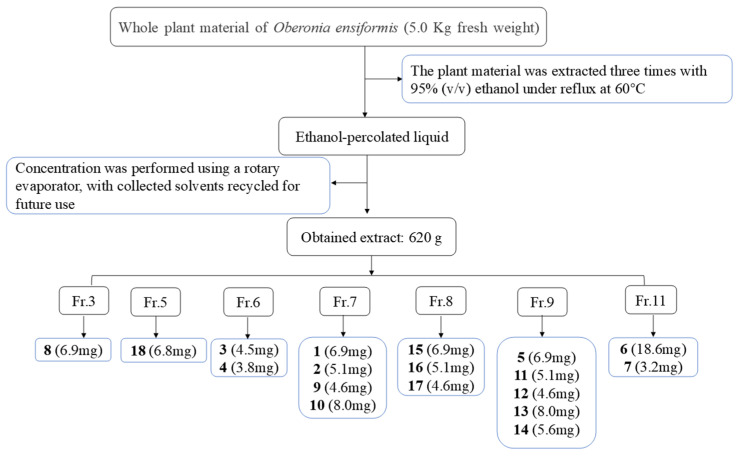
Isolation procedure of chemical constituents of *O*. *ensiformis*.

The *O. myosurus* whole herbs (5.0 kg) were air-dried, extracted three times with 95% EtOH (20 L × 3) at room temperature, and then filtered. The extracts were combined and concentrated to afford an organic extract (ca. 620 g), which was mixed with silica gel, loaded onto a silica gel column, and eluted with CH_2_Cl_2_-CH_3_OH (1:0, 100:1, 50:1, 20:1, 10:1, 5:1, 2:1, 1:1, and 0:1) to obtain 15 fractions (Fr. 1–12) (Figure 6.) Fraction 3 (16.7 g) was separated on a Sephadex LH-20 (CH_2_Cl_2_/MeOH 1:1) column, and Fraction C3-2 was further fractionated using silica gel CC and eluted with CHCl_3_–methanol (MeOH) (80:1–10:1, *v*/*v*) to obtain six subfractions (3–1–3–6). Subfraction 3–4 was isolated using Sephadex LH-20 CC (CH_2_Cl_2_/MeOH; 1:1) to obtain compound 8 (9.6 mg).

Fraction 5 (86 g) was further fractionated using silica gel CC and eluted with CHCl3–methanol (MeOH) (100:1–10:1, *v*/*v*) to obtain five subfractions (5–1–5–5). Subfraction 5–5 was isolated using Sephadex LH-20 CC (Sigma-Aldrich, St. Louis, MO, USA, CH_2_Cl_2_/MeOH; 1:1), repeated silica gel CC (CH_2_Cl_2_/MeOH; 50:1→10:1), and preparative TLC (petroleum ether/ethyl acetate; 10:1), resulting in the isolation of compound 18 (6.5 mg).

Fraction 6 (28 g) was subjected to column chromatography (MCI, MeOH/H_2_O 10% to 100%) and further fractionated using Sephadex LH-20 (CH_2_Cl_2_/MeOH 1:1), yielding six fractions (Fr.1-Fr.6). Fraction C3 (1.2 g) were separated by silica gel CC (CH_2_Cl_2_/MeOH from 50:1 to 10:1) and purified by MPLC (ODS, MeOH/H2O from 10% to 100%) to yield compounds **3** (4.5 mg) and **4** (3.8 mg).

Fraction seven (64 g) was further fractionated using silica gel CC and eluted with CHCl_3_–methanol (MeOH) (100:1–10:1, *v*/*v*) to obtain six subfractions (7–1–7–6). Subfraction 7–5 was isolated using Sephadex LH-20 CC (CH_2_Cl_2_/MeOH; 1:1), repeated silica gel CC (CH_2_Cl_2_/MeOH; 60:1→10:1), and preparative TLC (petroleum ether/ethyl acetate; 1:1), resulting in the isolation of compounds **1** (6.9 mg), 2 (5.1 mg), **9** (4.6 mg), and **10** (8 mg).

Fraction 8 (64 g) was subjected to silica gel CC (CH_2_Cl_2_/MeOH from 50:1 to 10:1) and Sephadex LH-20 (MeOH) to produce two fractions (C8-1 and C8-5). Fraction C8-1 was purified by HPLC (MeOH/H2O 65:35, 2.5 mL/min), and compounds **15** (62.7 mg, tR 14.6 min), **17** (9.1 mg tR 33.5 min), and **16** (8.2 mg tR 40.7 min) were obtained.

Fraction 9 (80 g) was further fractionated using silica gel CC and eluted with CHCl_3_–methanol (MeOH) (60:1–10:1, *v*/*v*) to obtain ten subfractions (9–1–9–10). Subfraction 9–3 was isolated using Sephadex LH-20 CC (CH2Cl2/MeOH; 1:1), repeated silica gel CC (CH_2_Cl_2_/MeOH; 100:1→10:1), MCI resin (MeOH–H_2_O, 100:0 → 1:1), and preparative TLC (petroleum ether/ethyl acetate; 1:1), resulting in the isolation of compounds **5** (28.2 mg), **11** (21.4 mg), **12** (39.3 mg), **13** (28.7 mg), and **14** (36.8 mg).

Fraction 11 (79 g) was separated sequentially using silica gel (CHCl_3_–MeOH, 100:0 → 10:1), MCI resin (MeOH–H_2_O, 100:0 → 1:1), and Sephadex LH-20 (MeOH) and then preparative HPLC to afford compounds **6** (18.6 mg) and **7** (3.2 mg).

### 4.4. Spectroscopic Data

Oberoniaensiformisin A (**1**): light-yellow oil; (+)-HRESIMS *m*/*z* 275.0901 [M + Na]^+^ (calcd. for C_13_H_16_O_5_Na, 275.0895); IR *ν*max:3396, 2922, 2851, 1747, 1552, 1435, 1384, 1224, 902, 772, 581, 473 cm^−1^, for ^1^H NMR (600 MHz, CDCl_3_) and ^13^C NMR (150 MHz, CDCl_3_); data shown in Table 1.

Oberoniaensiformisin B (**2**); light-yellow oil; (+)-HRESIMS *m*/*z* 281.1021 [M + H]^+^ (calcd. for C_14_H_17_O_6_, 281.1025); IR *ν*max: 3267, 2922, 2850, 1726, 1666, 1643, 1600, 1470, 1427, 1362, 1336, 1302, 1246,1208, 1095, 1035, 999, 975, 928, 796, 771, 675, 555, 470 cm^−1^, for ^1^H NMR (600 MHz, CDCl_3_) and ^13^C NMR (150 MHz, CDCl_3_); data are shown in Table 1.

Oberoniaensiformisin C (**3**); colorless oil; [α${]}_{D}^{25}$ −4.0 (c 0.1, MeOH); (+)-HRESIMS *m*/*z* 251.0892 [M + H]^+^ (calcd. for C_13_H_15_O_5_, 251.0919); IR *ν*max: 3399, 2923, 2852, 1601, 1553, 1442, 1360, 1240, 1067, 902, 791, 644, 480 cm^−1^. for ^1^H NMR (600 MHz, CDCl_3_) and ^13^C NMR (150 MHz, CDCl_3_); data are shown in Table 2.

Oberoniaensiformisin D (**4**); colorless oil; (+)-HRESIMS *m*/*z* 235.0979 [M + H]^+^ (calcd. for C_13_H_15_O_4_, 235.0970); IR *ν*max: 3433, 2926, 1638, 1384, 1104, 469 cm^−1^, for ^1^H NMR (600 MHz, MeOH) and _13_C NMR (150 MHz, MeOH); data are shown in Table 2.

Oberoniaensiformisin E (**5**); yellow amorphous powder; [α${]}_{D}^{25}$ −4.0 (c 0.1, MeOH); (+)-HRESIMS *m*/*z* 429.1902 [M + Na]^+^ (calcd. for C_22_H_30_O_7_Na, 429.1889); IR *ν*max: 3428, 2926, 1712, 1602, 1555, 1453, 1355, 1304, 1202, 1102, 1061, 771 cm^−1^, for ^1^H NMR (600 MHz, DMSO) and ^13^C NMR (150 MHz, DMSO); data are shown in Table 3.

Oberoniaensiformisin F (**6**); yellow amorphous powder; [α${]}_{D}^{25}$ −14.0 (c 0.1, MeOH); (+)-HRESIMS *m*/*z* 447.2022 [M + H]^+^ (calcd. for C_24_H_31_O_8_, 447.2019); IR *ν*max: 3855, 3752, 3737, 3553, 3415, 2921, 2850, 1638, 1618, 1030, 623, 481 cm^−1^, for ^1^H NMR (600 MHz, DMSO-*d*_6_) and ^13^C NMR (150 MHz, DMSO-*d*_6_); data are shown in Table 4.

Oberoniaensiformisin G (**7**); light-yellow oil; [α${]}_{D}^{25}$ −78.7 (c 0.1, MeOH); (+)-HRESIMS *m*/*z* 445.2249 [M + H]^+^ (calcd. for C_25_H_33_O_7_, 445.2226); IR *ν*max: 3394, 2922, 2850, 1716, 1552, 1436, 1354, 1258, 1107, 1100, 902, 770, 646, 473 cm^−1^, for ^1^H NMR (600 MHz, MeOH) and ^13^C NMR (150 MHz, MeOH); data are shown in Table 4.

### 4.5. Quantum Chemical Calculations

Conformational analysis was initially performed using the GMMX module in GaussView with the MMFF94 force field, considering all conformers within a 3.5 kcal/mol energy window. The resulting low-energy conformers were further optimized at the B3LYP/6-311+G(2d,p) level of theory using Gaussian 16. To be compared with experimental data, electronic circular dichroism (ECD) spectra were calculated at the TDDFT/B3LYP/6-311+G(2d,p) level in MeOH, while NMR chemical shifts were calculated at the mPW1PW91/6-311+G(d,p) level in CHCl_3_. Finally, the theoretical values were Boltzmann-averaged based on the relative populations of the optimized conformers and compared with experimental observations. The DP4+ probability analysis was performed on the calculated ¹³C NMR chemical shifts to assess the confidence level of each proposed stereochemical configuration [18,19].

### 4.6. Antibacterial Assay

In accordance with the background of folk medicine application of *O. myosurus*, all isolated compounds were evaluated for their potential antibacterial activity against the three bacterial strains *Escherichia coli* ATCC25922, *Staphylococcus aureus subsp. aureus* ATCC29213, and *Pseudomonas aeruginosa* ATCC27853. Ciprofloxacin was used as a positive control. Targeted microbes were cultivated in LB medium (yeast extract 5 g/L, peptone 10 g/L, NaCl 10 g/L, pH = 7.4) overnight at 37 °C and diluted bacterial suspension (5 × 10^5^ CFU/mL) for the test. The minimum inhibitory concentrations (MICs) of samples and positive control were determined in sterile 96-well plates by the modified broth dilution test [20,21]. All wells were filled with 196 μL of bacterial suspension containing 5 × 10^5^ CFU/mL. Test samples (4 μL) with concentrations of serial dilutions were added to each well. The negative control was a medium containing 1% DMSO, and the positive control used was ciprofloxacin. The final concentrations of test compounds were 128, 64, 32, 16, 8, 4, 2, 1, and 0.5 μg/mL in medium. After incubation, the minimum inhibitory concentration (MIC) was defined as the lowest test concentration that completely inhibited the growth of the test organisms.

### 4.7. Antioxidant Assay

The activity screening of 18 compounds isolated from *O. ensiformis* was carried out using 96-well plates and an enzyme labeler with DPPH methanol solution as substrate (0.15 mmol/L) and sodium ascorbate as positive control [22]. The gradient mass concentration of the samples to be tested was 200, 100, 50, 25, 12.5 μg/mL and then divided into a sample group (100 μL DPPH solution + 100 μL sample, A), a sample control group (100 μL methanol + 100 μL sample, A_0_), and a negative control (100 μL DPPH solution + 100 μL methanol, A_1_). The samples to be tested were fully reacted under light-avoiding conditions, and the OD values were determined at 517 nm after 30 min [23]. The experiments were performed three times in parallel, and the DPPH radical scavenging rate was calculated. The scavenging rate of DPPH radical was calculated according to the following equation [24].

E = [1 − (A − A_a_)/A_b_] × 100%

A:100 μL of test compounds or L-Ascorbic Acid Sodium Salt and 100 μL of DPPH.

A_a_: 100 μL of test compounds or L-Ascorbic Acid Sodium Salt and 100 μL of MeOH.

A_b_: 100 μL of MeOH and 100 μL of DPPH.

### 4.8. α-Glucosidase Inhibition Assay

The inhibitory activity of the obtained compounds on α-glucosidase was evaluated using the method mentioned in previous studies with minor modifications [25,26,27]. The *α*-glucosidase inhibitor acarbose was used as a positive control, and the total experimental system was 200 µL. The following sentences describe the assay process in brief: The compounds were first dissolved in DMSO, then diluted with 0.1 M phosphate buffer (PBS, pH 6.8, DMSO concentration lower than 1%); 50 μL compounds or acarbose (dissolved in 0.1 M PB, pH 6.8) with different concentrations were mixed with 20 μL of 0.2 U/mL *α*-glucosidase (dissolved in 0.1 M PB, pH 6.8), and then incubated at 37 °C for 10 min. Subsequently, 50 μL of 5.0 mM *p*-Nitrophenyl *α*-D-glucopyranoside (pNPG, dissolved in 0.1 M PB, pH 6.8) was added to the mixture. After incubation at 37 °C for 30 min, the reaction was stopped by adding 80 μL of 0.2 M sodium carbonate solution (dissolved in ultrapure water) to the reaction system. The absorbance value was measured at 405 nm using a microplate reader. The inhibitory activity of compounds or acarbose on *α*-glucosidase was calculated by the formula

Inhibition rate (%) = 1 − (A_a_ − A_b_)/A_c_ − A_d_ × 100

A_a_: Absorbance of the reaction system containing 20 μL of test compounds or acarbose at different concentrations, 50 μL of *α*-glucosidase, and 50 μL of pNPG.

A_b_: Absorbance of the reaction system containing 70 μL of test compounds or acarbose at different concentrations and 50 μL of *α*-glucosidase.

A_c_: Absorbance of the reaction system containing 20 μL PBS, 50 μL *α*-glucosidase, and 50 μL pNPG.

A_d_: Absorbance of the reaction system containing 70 μL of PBS and 50 μL of *α*-glucosidase.

## 5. Conclusions

In conclusion, our investigation led to the identification of 18 tandem prenylated *p*-hydroxybenzoic acid derivatives from *O. ensiformis*, including 7 previously undescribed compounds (**1**–**7**). The intricate stereochemical configurations of these isolates were successfully determined through comprehensive spectroscopic analyses, complemented by comparative studies between experimental and calculated ECD spectra. In addition, all the compounds were investigated for their biological activities, and in the antioxidant assay, compounds **6** and **12** showed scavenging of DPPH radicals above 50%, with IC_50_ values of 173.76 ± 23.92 and 185.36 ± 1.96, respectively. The in vitro hypoglycemic activity of the obtained compounds was evaluated using an *α*-glucosidase inhibition assay. The results demonstrate that some of the compounds exhibit varying degrees of inhibitory activity against *α*-glucosidase, with IC_50_ values ranging from 34.03 to 106.10 μg/mL. The current study significantly expands our understanding of the in vitro biological activities associated with isoprenylated *p*-hydroxybenzoic acid derivatives. While these findings establish a solid theoretical foundation for further research on *O. ensiformis*, additional investigations are warranted to explore potential alternative biological activities of the isolated compounds and to validate their pharmacological relevance.

## Figures and Tables

**Table 1:** ^1^H (600 MHz) and ^13^C (150 MHz) NMR data for compounds **1** and **2**.

Position	1		2	
	*δ*_C_	*δ*_H_ (*J* in Hz)	*δ*_C_	*δ*_H_ (*J* in Hz)
1	126.7		119.1	
2	115.2	7.49, d (2.1)	150.0	
3	148.9	7.43, d (2.1)	142.8	
4	149.2		150.0	
5	134.1		123.7	
6	122.9		121.5	7.69, s
7	166.6		165.3	
8				
9				
1′	23.7	2.93, t (7.8)	137.1	7.73, d (16.4)
2′	47.6	2.79, t (7.8)	128.5	6.77, d (16.4)
3′	207.5		198.6	
4′	30.0	2.17, s	27.7	2.39, s
COOCH_3_	52.1	3.88, s	52.5	3.93, s
OCH_3_-2			62.7	3.95, s
OCH_3_-4	61.3	3.84, s	61.5	4.00, s

Measured in CDCl_3_ (*δ*_H_ 7.26 ppm, *δ*_C_ 77.0 ppm).

**Table 2:** ^1^H (600 MHz) and ^13^C (150 MHz) NMR data for compounds **3** and **4**.

Position	3 ^a^		4 ^b^	
	*δ*_C_	*δ*_H_ (*J* in Hz)	*δ*_C_	*δ*_H_ (*J* in Hz)
2	92.7	4.85, d (1.0)	167.0	
3	76.7	5.12, br s	101.8	6.77, d (1.0)
4	114.3	7.01, s	124.4	8.28, d (1.8)
5	136.6		126.2	
6	157.0		126.7	7.98, dd (8.6, 1.8)
7	109.5	7.30, s	111.9	7.54, d (8.6)
8	152.3		158.8	
9	113.3		130.1	
1′	141.4		69.7	
2′	113.0	4.94, br s	28.9	1.65, s
		5.10, br s		
3′	17.6	1.75, s	28.9	1.65, s
COOCH_3_	52.6	3.95, s	52.6	3.94, s
-C=O	170.3		168.8	

^a^ Measured in CDCl_3_ (*δ*_H_ 7.26 ppm, *δ*_C_ 77.0 ppm). ^b^ Measured in CD_3_OD (*δ*_H_ 3.31 ppm, *δ*_C_ 49.0 ppm).

**Table 3:** ^1^H (600 MHz) and ^13^C (150 MHz) NMR data for compound **5a**–**5b**.

Position	5a		5b	
	*δ*_C_	*δ*_H_ (*J* in Hz)	*δ*_C_	*δ*_H_ (*J* in Hz)
1	120.6		120.6	
2	129.4	7.58, s	129.4	7.58, s
3	128.4		128.3	
4	158.0		158.1	
5	125.9		126.0	
6	130.9	7.60, s	131.0	7.59, s
7	165.1		165.1	
1′	28.2	3.26, d (6.9)	28.2	3.26, d (6.9)
2′	122.4	5.24, t (7.4)	122.4	5.24, t (7.4)
3′	131.9		131.9	
4′	25.5	1.70, s	25.5	1.70, s
5′	17.7	1.69, s	17.7	1.68, s
1″	37.7	2.80, d (6.6)	37.7	2.80, d (6.6)
2″	74.9	4.23, d (18.8)	74.7	4.23, d (18.8)
3″	147.1		147.2	
4″	110.4	4.75, d (3.7)	110.3	4.75, d (3.7)
		4.90, d (6.2)		4.90, d (6.2)
5″	18.0	1.73, s	18.1	1.73, s
1‴	175.8		175.8	
2‴	75.3		75.3	
3‴	74.2	5.09, d (6.4)	74.2	5.09, d (6.4)
4‴	13.6	1.22, d (6.3)	13.6	1.22, d (6.3)
5‴	22.3	1.27, s	22.3	1.27, s

Measured in DMSO-*d*_6_ (*δ*_H_ 2.49 ppm, *δ*_C_ 39.5 ppm).

**Table 4:** ^1^H (600 MHz) and ^13^C (150 MHz) NMR data for compounds **6** and **7**.

Position	6 ^a^		7 ^b^	
	*δ*_C_	*δ*_H_ (*J* in Hz)	*δ*_C_	*δ*_H_ (*J* in Hz)
1	120.3		122.1	
2	129.1	7.49, s	129.9	7.57, s
3	128.2		129.5	
4	158.1		158.7	
5	126.1		129.5	
6	130.7	7.54, s	129.9	7.57, s
7	165.3		167.7	
1′	28.0	3.25, d (7.6)	29.2	3.30, d (1.6)
2′	122.1	5.23, t (7.6)	122.8	5.30, m
3′	132.2		134.3	
4′	25.4	1.68, s	25.9	1.76, s
5′	15.6	1.64, s	17.8	1.70, s
1″	37.4	2.75, d (4.3) 2.80, d (8.3)	29.2	3.30, d (1.6)
2″	74.6	4.19, dd (8.3)	122.8	5.30, m
3″	147.1		134.3	
4″	18.1	4.88, s	25.9	1.77, s
		4.73, s		
5″	110.2	1.71, s	17.8	1.70, s
1‴	128.0		129.3	
2‴	138.8	6.68, br s	139.5	6.86, br s
3‴	65.5	4.27, br s	67.4	4.41, br s
4‴	67.5	3.79, s	69.2	3.98, dd (4.4)
5‴	70.2	5.15, dt (5.9)	71.5	5.34, dt (4.3)
6‴	27.4	2.21, br dd (18.3)	28.3	2.41, dd (19.5)
		2.62, dd (18.3)		2.85, dd (18.5, 2.4)
7‴	167.7		168.3	
COOCH_3_			52.4	3.76, s

^a^ Measured in DMSO-*d*_6_ (*δ*_H_ 2.49 ppm, *δ*_C_ 39.5 ppm). ^b^ Measured in CD_3_OD (*δ*_H_ 3.31 ppm, *δ*_C_ 49.0 ppm).

**Table 5:** Clearance rate of compounds on DPPH (mean ± SD).

No.	Compound	IC_50_ (μg/mL) *
positive control	L-ascorbate	2.37 ± 0.11
**6**	oberoniaensiformisin F	173.76 ± 23.92
**12**	nervogenic acid	185.36 ± 1.96

* IC_50_: Clearance rate of DPPH 50% activity (concentration in µg/mL required for a 50% reduction in antioxidant activity).

**Table 6:** Inhibitory effects of compounds on α-glucosidase (mean ± SD).

No.	Compound	IC_50_ (μg/mL) *
positive control	acarbose	0.66 ± 0.36
**5**	oberoniaensiformisin F	106.10 ± 2.12
**6**	oberoniaensiformisin F	58.07 ± 6.22
**12**	nervogenic acid	34.03 ± 0.16
**13**	oberoniamyosurusin L	86.53 ± 1.08
**15**	oberoniamyosurusin I	38.80 ± 4.43

* IC_50_: inhibition of α-glucosidase 50% activity (concentration in µg/mL required for a 50% reduction in α-glucosidase-inhibitory activity).

## Data Availability

Data are contained within the article and Supplementary Materials.

## Supplementary Materials

The following supporting information can be downloaded at https://www.mdpi.com/article/10.3390/molecules30102132/s1: Pages 4–27: *1*H NMR, *13*C NMR, *1*H-*1*H COSY, HSQC, HMBC, NOESY HRESIMS, and IR spectrum of compounds **1**–**7**. Page 28: Enzyme inhibition results of inactive compounds among compounds **1–18**. Page 29: Configuration determination materials for compound **3**.
