# Supplementary material for: Antimicrobial, Antioxidant, and α-Glucosidase-Inhibitory Activities of Prenylated p-Hydroxybenzoic Acid Derivatives from Oberonia ensiformis
Source: Molecules. 2025 May 12;30(10):2132. doi: 10.3390/molecules30102132 (PMC12114510; doi:10.3390/molecules30102132)
Supplement: Supplementary file 1 [file molecules-30-02132-s001.zip › molecules-3590090-supplementary.pdf]

Antimicrobial, Antioxidant, and *α*-Glucosidase-Inhibitory Activities of Prenylated *p*-Hydroxybenzoic Acid Derivatives from *Oberonia ensif*

Lu-Lu Wang†, Wei Tang†, Zhuo Wang, Yi-Xiang Wang, Ning Li* and Fu-Cai Ren*

School of Pharmacy, Anhui Medical University, Hefei 230032, China

* Correspondence: 1993500019@ahmu.edu.cn (N.L.); renfucai@ahmu.edu.cn (F.-C.R.); Tel.: +86-551-65172133 (F.-C.R.)

† These authors contributed equally to this work.

Content List

S1. ^{1}H NMR spectrum (600 MHz, $CDCl_3$) of oberoniaensiformisin A 4

S2. ^{13}C NMR spectrum (150 MHz, $CDCl_3$) of oberoniaensiformisin A 4

S3. HSQC spectrum (600 MHz, $CDCl_3$) of oberoniaensiformisin A 5

S4. HMBC spectrum (600 MHz, $CDCl_3$) of oberoniaensiformisin A 5

S5. HRESIMS spectrum of oberoniaensiformisin A 6

S6. IR spectrum of oberoniaensiformisin A 6

S7. ^{1}H NMR spectrum (600 MHz, $CDCl_3$) of oberoniaensiformisin B 7

S8. ^{13}C NMR spectrum (150 MHz, CDCl3) of oberoniaensiformisin B 7

S9. HMBC spectrum (600 MHz, $CDCl_3$) of oberoniaensiformisin B 8

S10. HSQC spectrum (600 MHz, $CDCl_3$) of oberoniaensiformisin B 8

S11. HRESIMS spectrum of oberoniaensiformisin B 9

S12. IR spectrum of oberoniaensiformisin B 9

S13. ^{1}H NMR spectrum (600 MHz, $CDCl_3$) of oberoniaensiformisin C 10

S14. ^{13}C NMR spectrum (150 MHz, $CDCl_3$) of oberoniaensiformisin C 10

S15. HMBC spectrum (600 MHz, $CDCl_3$) of oberoniaensiformisin C 11

S16. HSQC spectrum (600 MHz, $CDCl_3$) of oberoniaensiformisin C 11

S17. HRESIMS spectrum of oberoniaensiformisin C 12

S18. IR spectrum of oberoniaensiformisin C 12

S19. ^{1}H NMR spectrum (600 MHz, CD_3OD) of oberoniaensiformisin D 13

S20. ^{13}C NMR spectrum (150 MHz, CD_3OD) of oberoniaensiformisin D 13

S21. HMBC spectrum (600 MHz, CD_3OD) of oberoniaensiformisin D 14

S22. HSQC spectrum (600 MHz, CD_3OD) of oberoniaensiformisin D 14

S23. HRESIMS spectrum of oberoniaensiformisin D 15

S24. IR spectrum of oberoniaensiformisin D 15

S25. ^{1}H NMR spectrum (600 MHz, DMSO-d_6) of oberoniaensiformisin E 16

S26 ^{13}C NMR spectrum (150 MHz, DMSO-d_6) of oberoniaensiformisin E 16

S27. HMBC spectrum (600 MHz, DMSO-d_6) of oberoniaensiformisin E (5) 17

S28. HSQC spectrum (600 MHz, DMSO-d_6) of oberoniaensiformisin E......................................17

S29. COSY spectrum (600 MHz, DMSO-d_6) of oberoniaensiformisin E......................................18

S30. HRESIMS spectrum of oberoniaensiformisin E..18

S.31. IR spectrum of oberoniaensiformisin E 19

S32. HPLC chromatogram of oberoniaensiformisin E 19

S33. ^{1}H NMR spectrum (600 MHz, DMSO-d_6) of oberoniaensiformis F 20

S34. ^{13}C NMR spectrum (150 MHz, DMSO-d_6) of oberoniaensiformisin F 20

S35. HMBC spectrum (600 MHz, DMSO-d_6) of oberoniaensiformisin F.....................................21

S36. HSQC spectrum (600 MHz, DMSO-d_6) of oberoniaensiformisin F......................................21

S37. COSY spectrum (600 MHz, DMSO-d_6) of oberoniaensiformisin F 22

S38. HRESIMS spectrum of oberoniaensiformisin F 22

S39. IR spectrum of oberoniaensiformisin F 23

S40. HPLC chromatogram of oberoniaensiformisin F...23

S41. ^{1}H NMR spectrum (600 MHz, CD_3OD) of oberoniaensiformisin G 24

S42. ^{13}C NMR spectrum (150 MHz, CD_3OD) of oberoniaensiformisin G....................................24

S43. HMBC spectrum (600 MHz, CD_3OD) of oberoniaensiformisin G...25

S44. HSQC spectrum (600 MHz, CD_3OD) of oberoniaensiformisin G..25

S45. COSY spectrum (600 MHz, CD_3OD) of oberoniaensiformisin G..26

S46. NOESY spectrum (600 MHz, CD_3OD) of oberoniaensiformisin G 26

S47. HRESIMS spectrum of oberoniaensiformisin G 27

S48. IR spectrum of oberoniaensiformisin G...27

S49. Enzyme inhibition results of inactive compounds among compounds **1**–**18**. 28

S50. Configuration identification of compound **3**:...28

S1. ^{1}H NMR spectrum (600 MHz, $CDCl_3$) of oberoniaensiformisin A

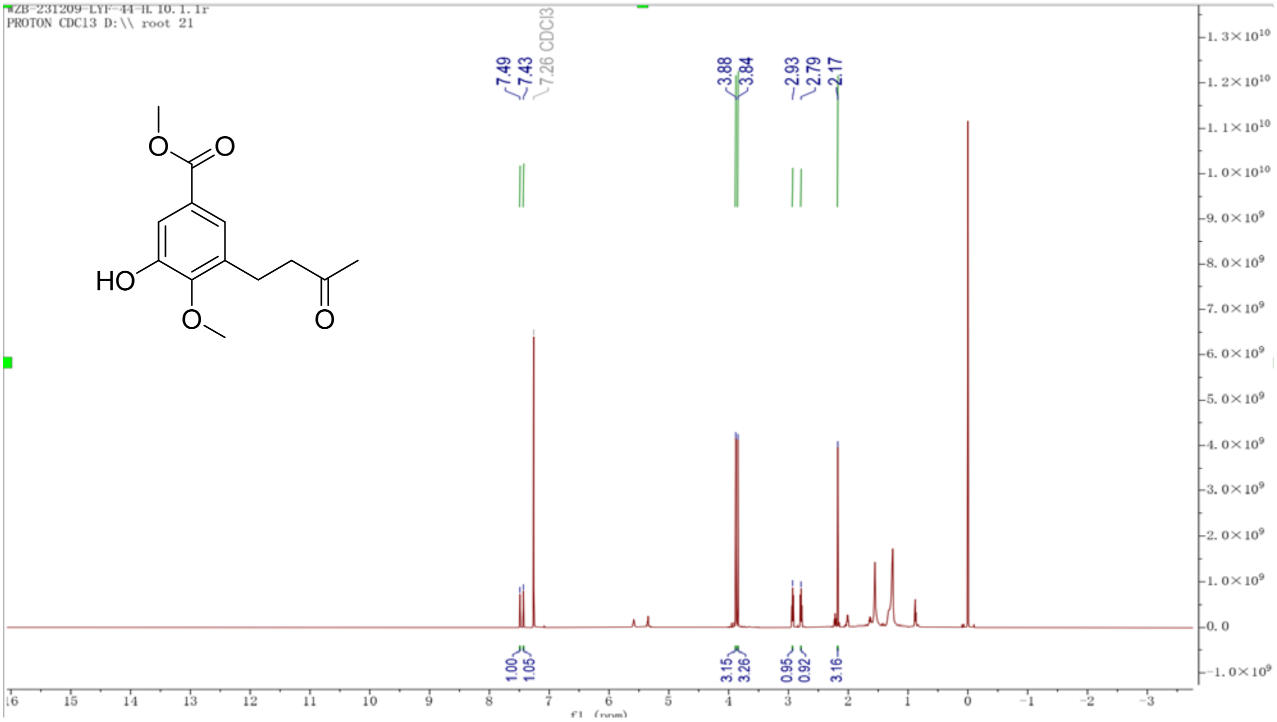

S2. ^{13}C NMR spectrum (150 MHz, $CDCl_3$) of oberoniaensiformisin A

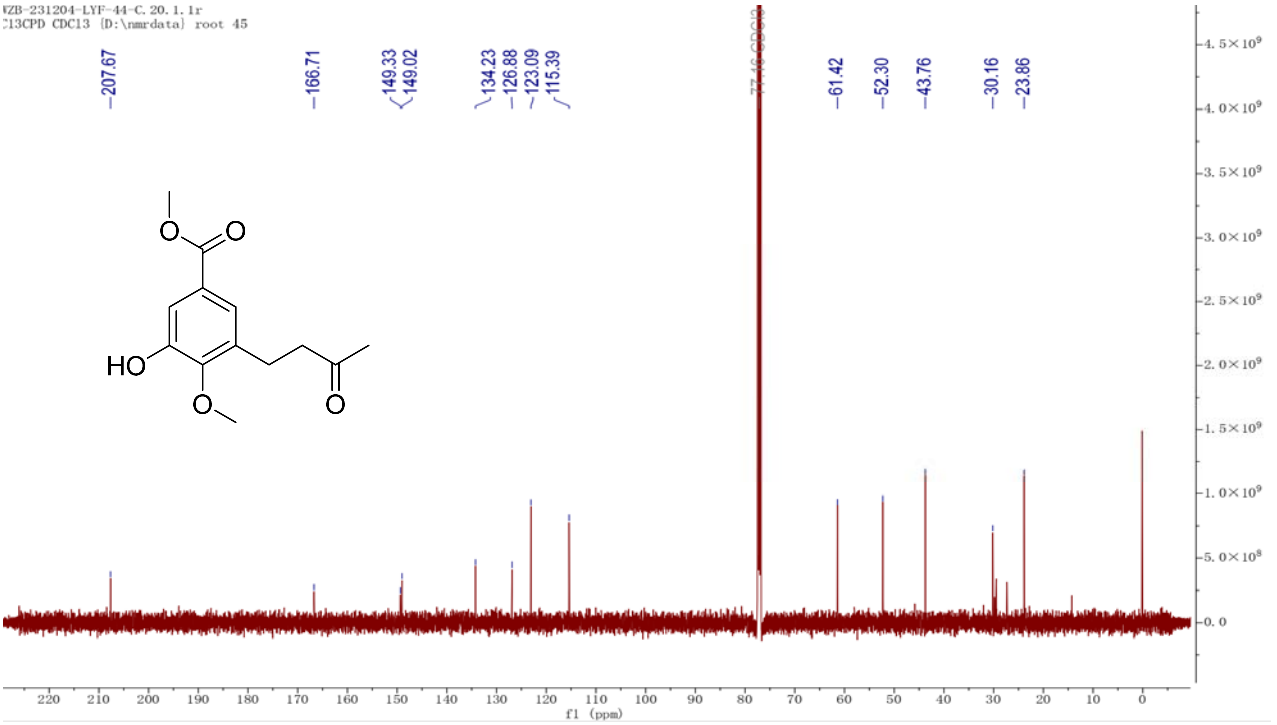

S3. HSQC spectrum (600 MHz, $CDCl_3$) of oberoniaensiformisin A

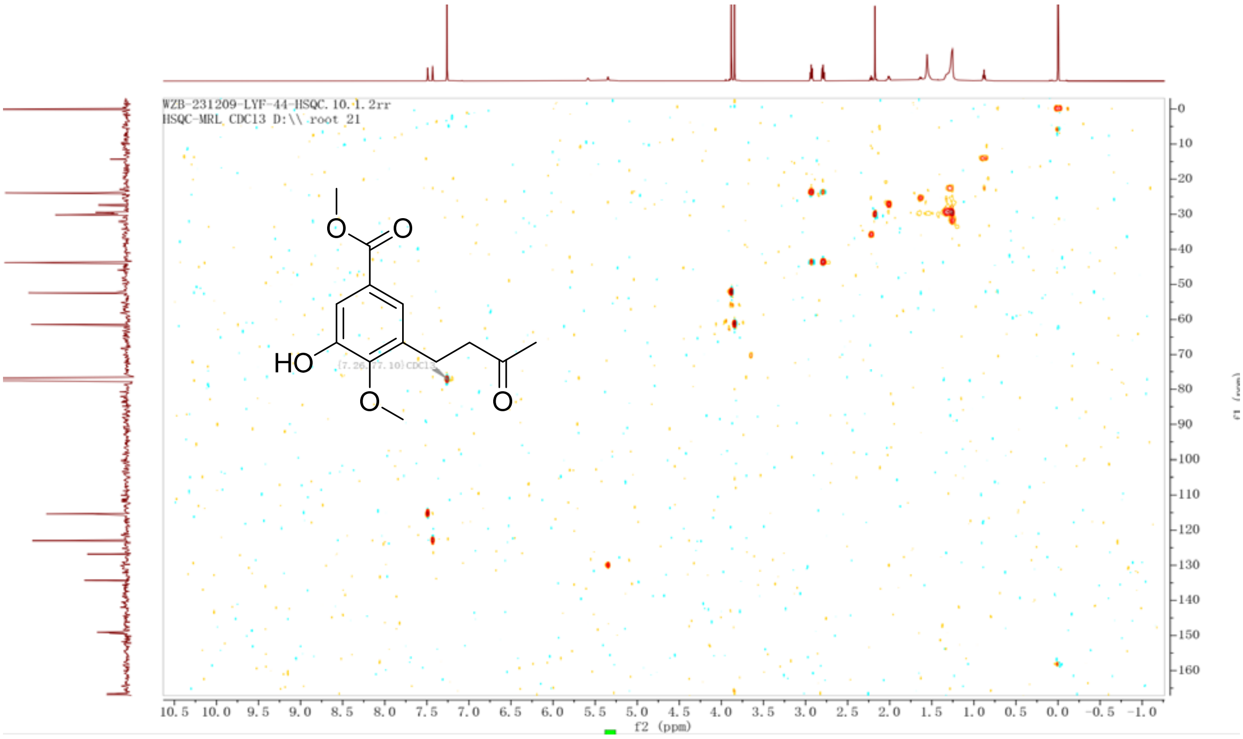

S4. HMBC spectrum (600 MHz, $CDCl_3$) of oberoniaensiformisin A

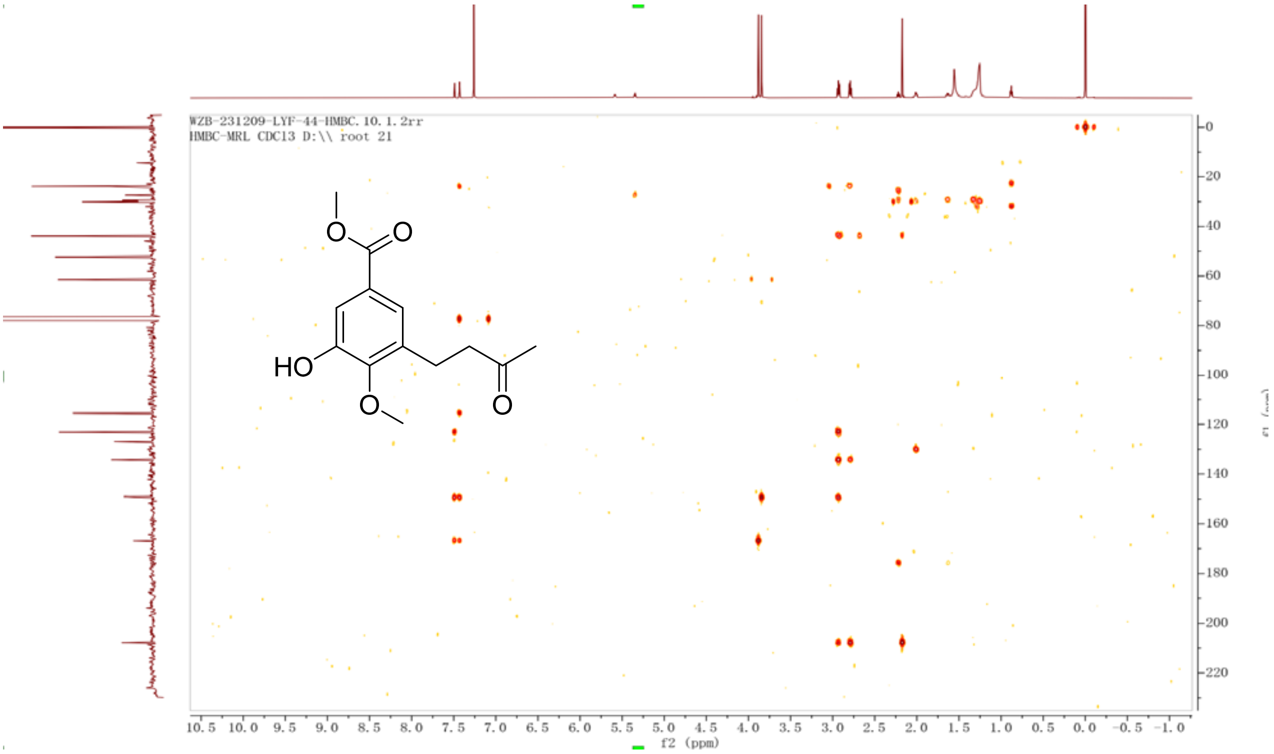

S5. HRESIMS spectrum of oberoniaensiformisin A

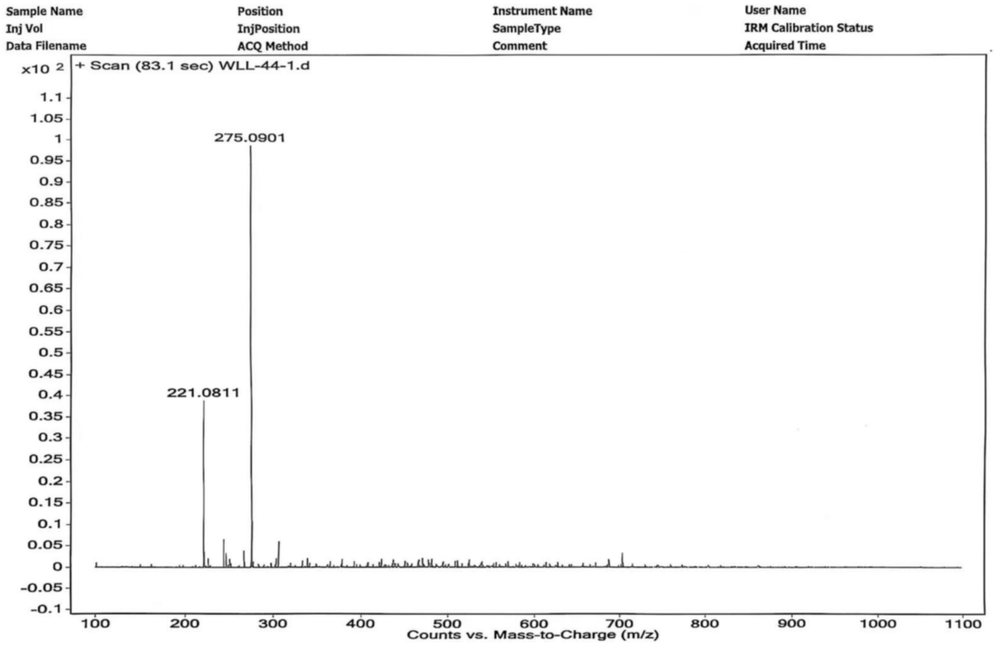

S6. IR spectrum of oberoniaensiformisin A

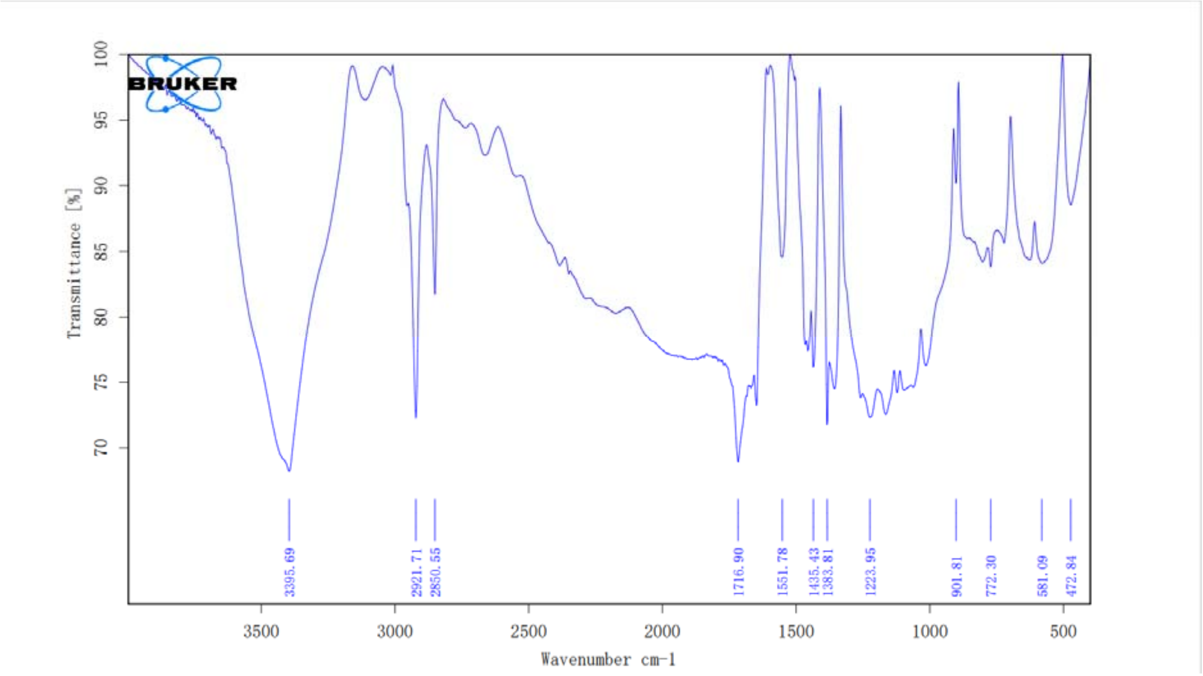

S7. ^{1}H NMR spectrum (600 MHz, $CDCl_3$) of oberoniaensiformisin B

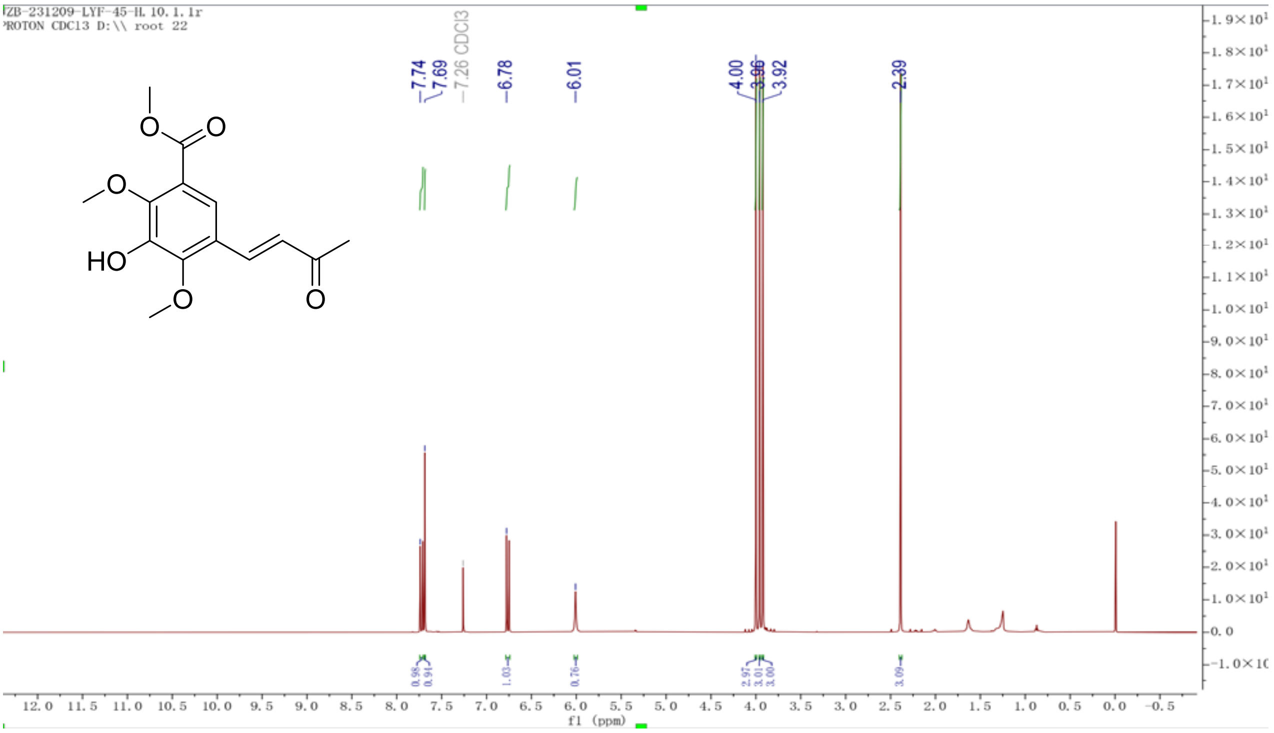

S8. ^{13}C NMR spectrum (150 MHz, CDCl3) of oberoniaensiformisin B

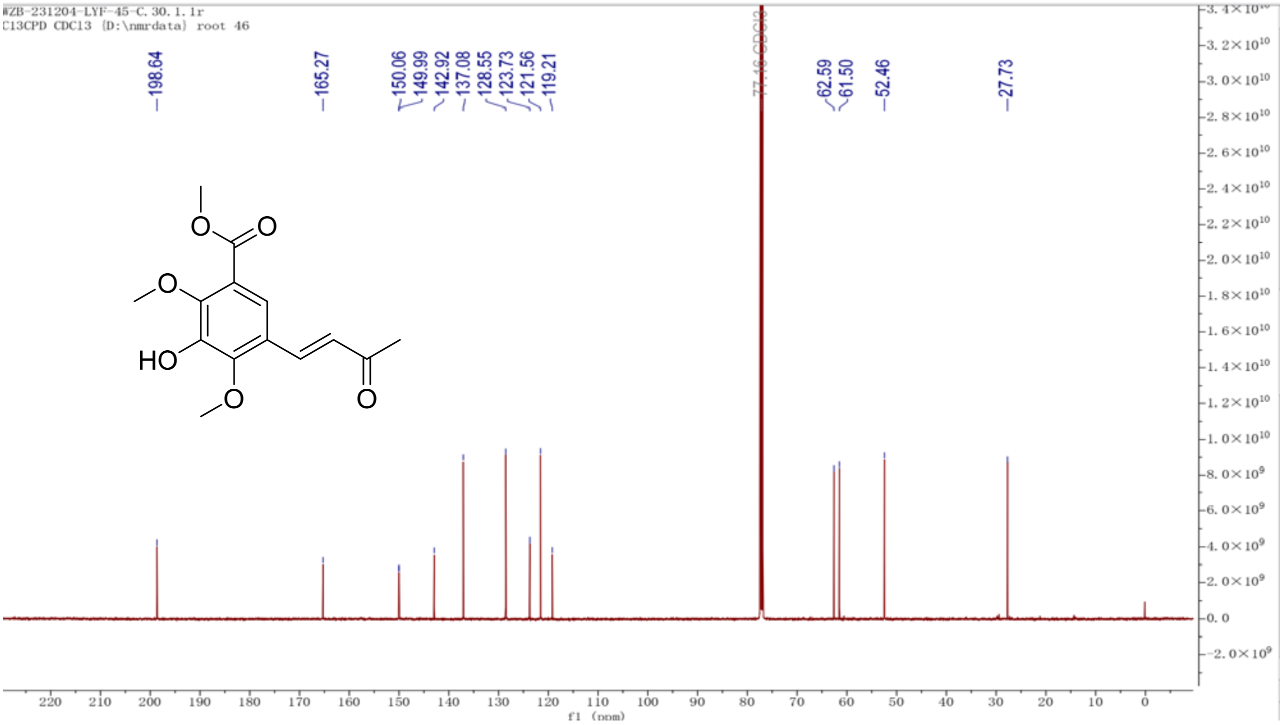

S9. HMBC spectrum (600 MHz, $CDCl_3$) of oberoniaensiformisin B

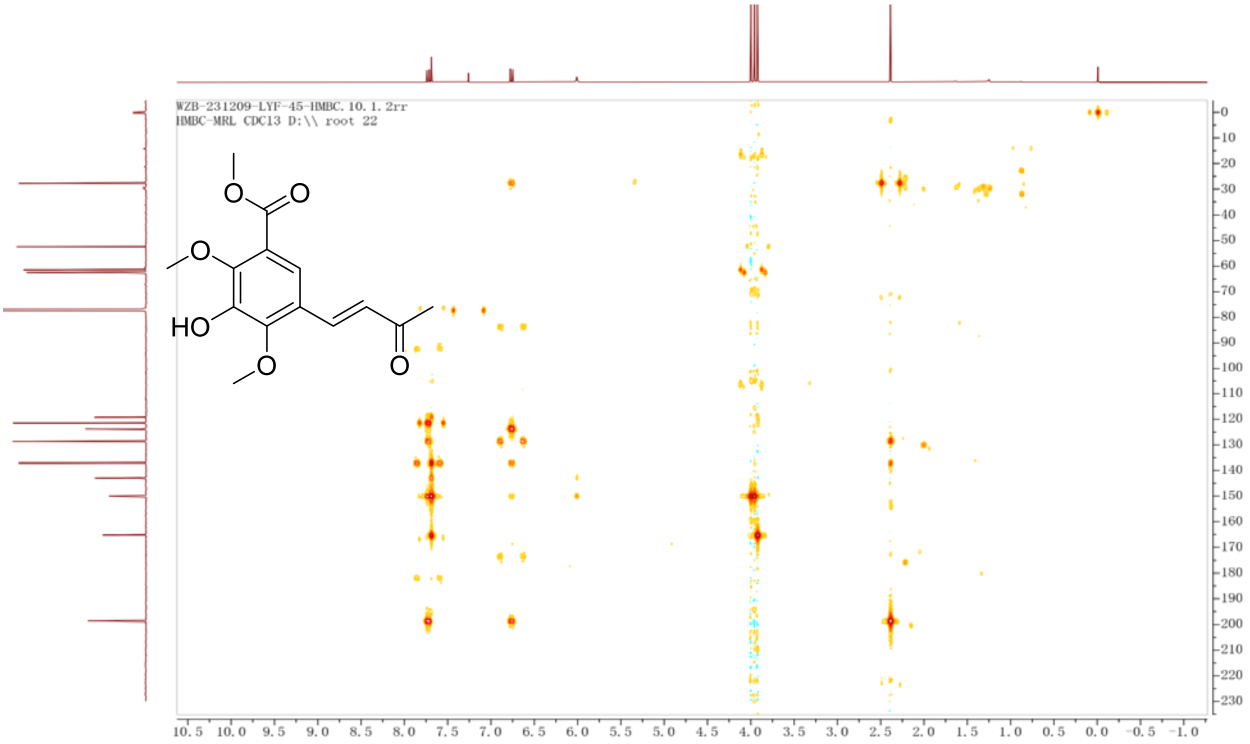

S10. HSQC spectrum (600 MHz, $CDCl_3$) of oberoniaensiformisin B

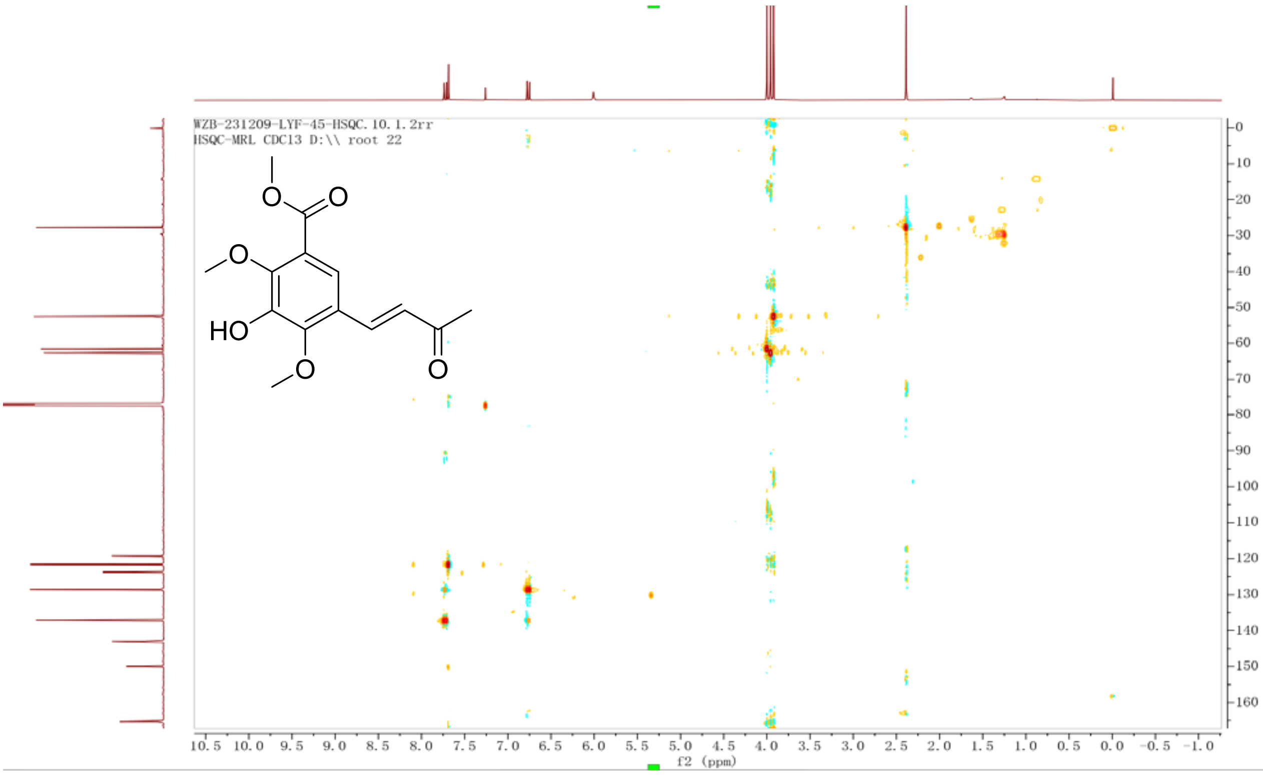

S11. HRESIMS spectrum of oberoniaensiformisin B

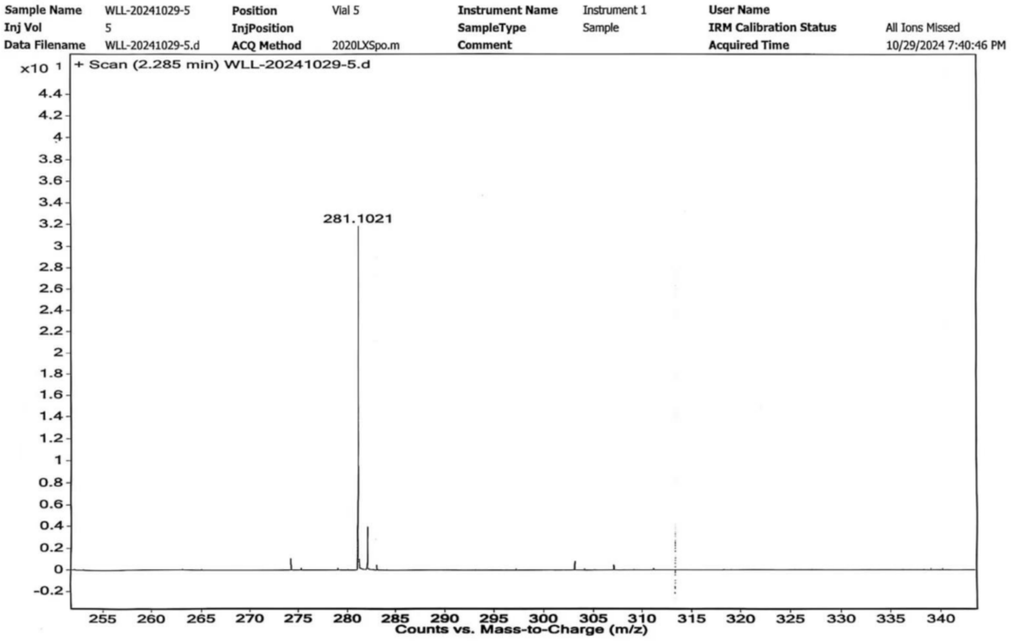

S12. IR spectrum of oberoniaensiformisin B

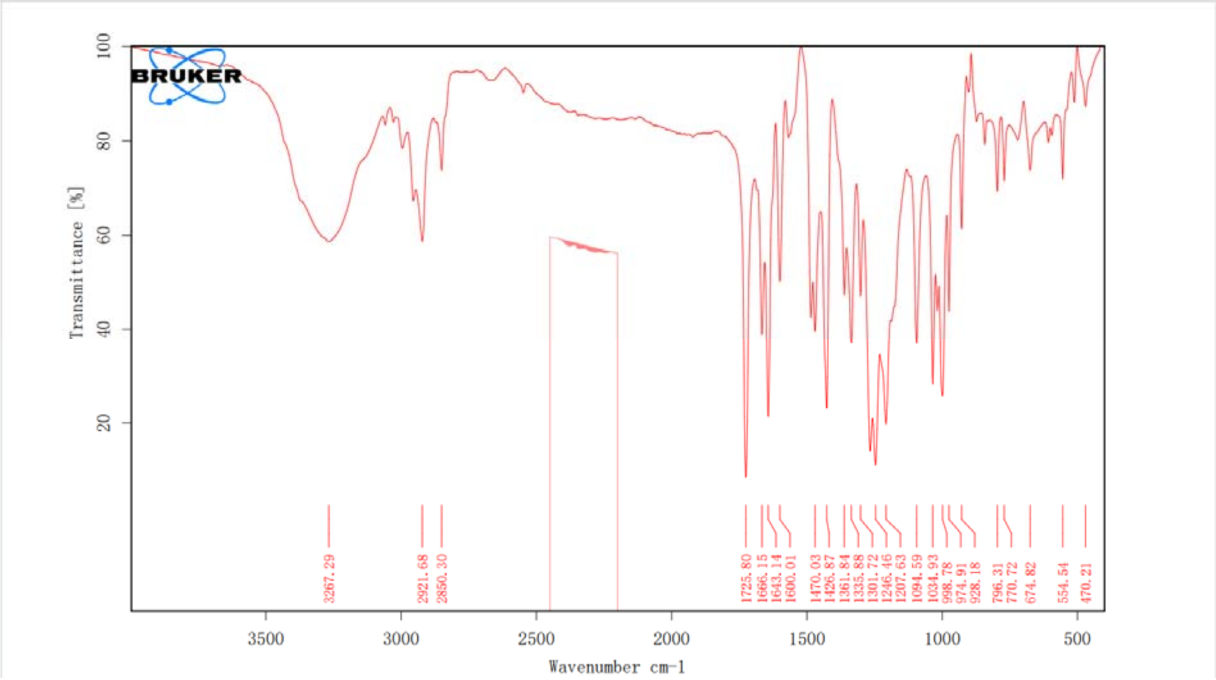

S13. ^{1}H NMR spectrum (600 MHz, $CDCl_3$) of oberoniaensiformisin C

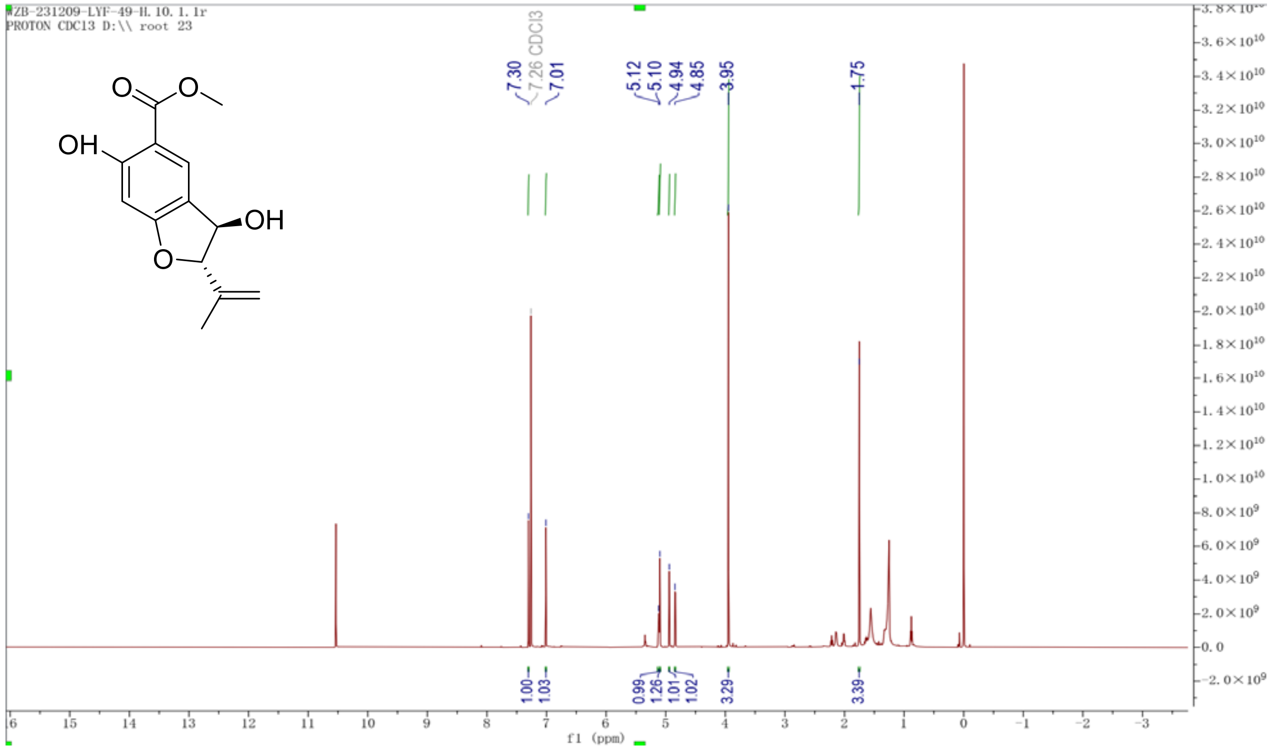

S14. ^{13}C NMR spectrum (150 MHz, $CDCl_3$) of oberoniaensiformisin C

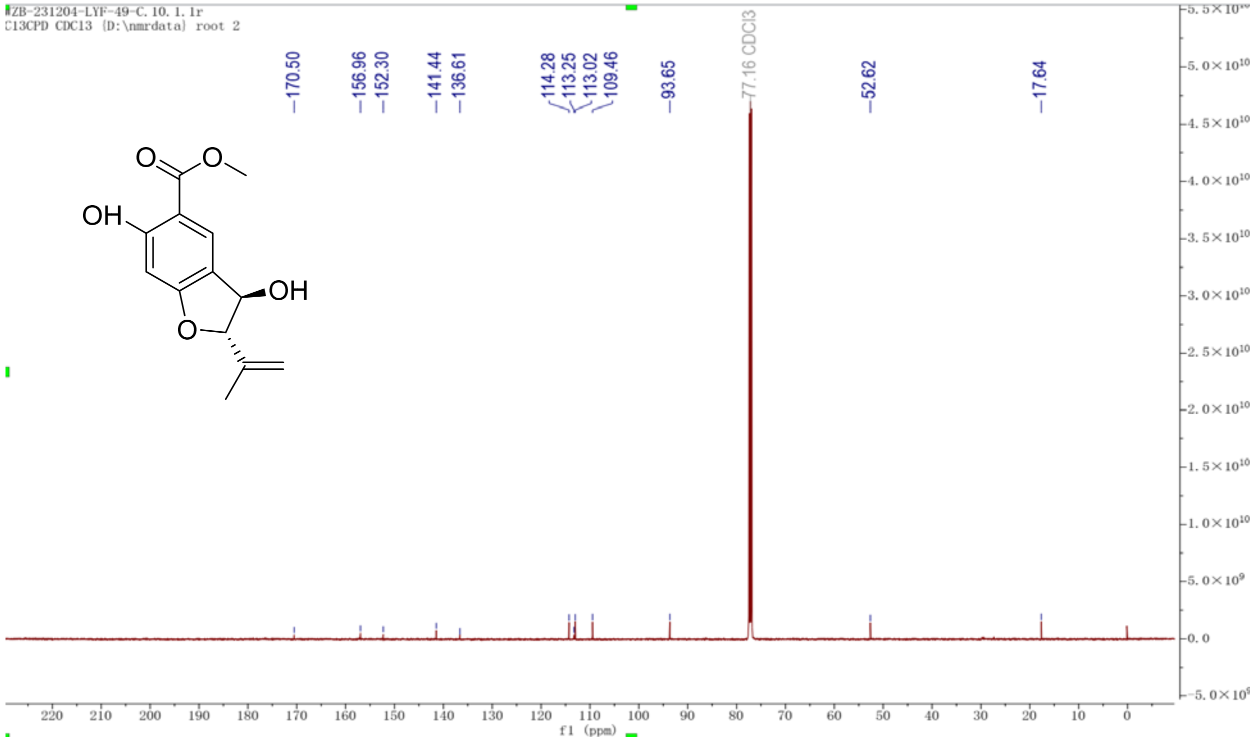

S15. HMBC spectrum (600 MHz, $CDCl_3$) of oberoniaensiformisin C

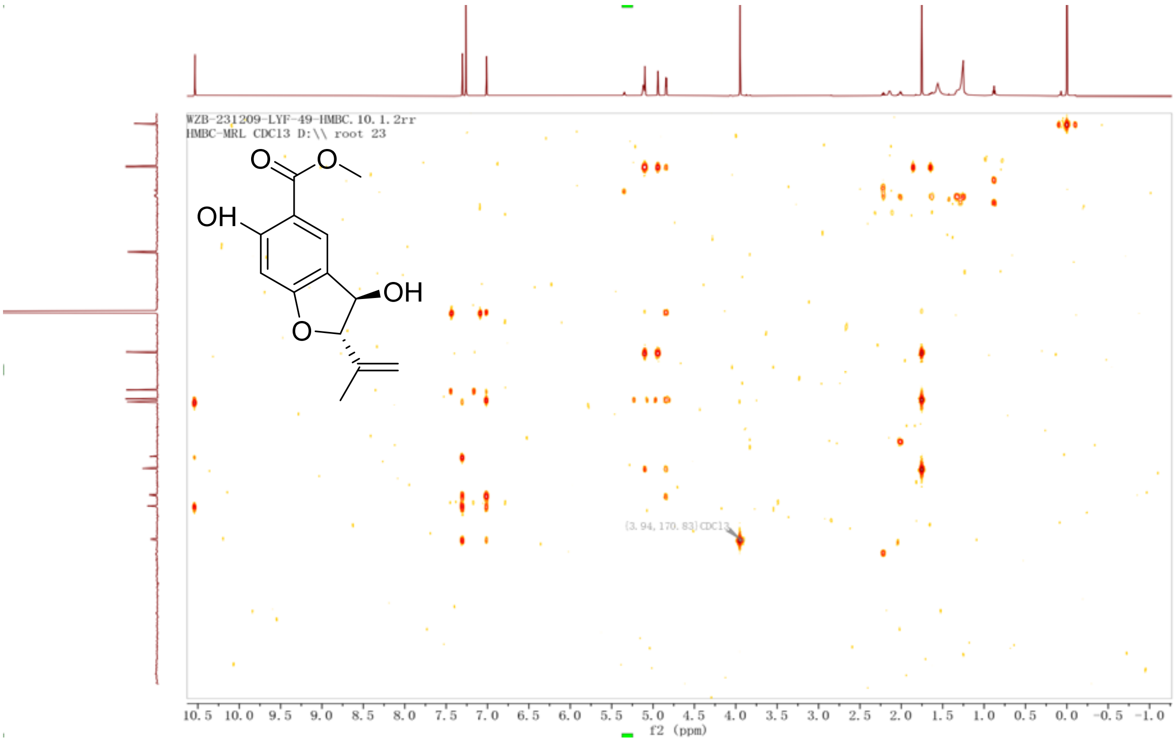

S16. HSQC spectrum (600 MHz, $CDCl_3$) of oberoniaensiformisin C

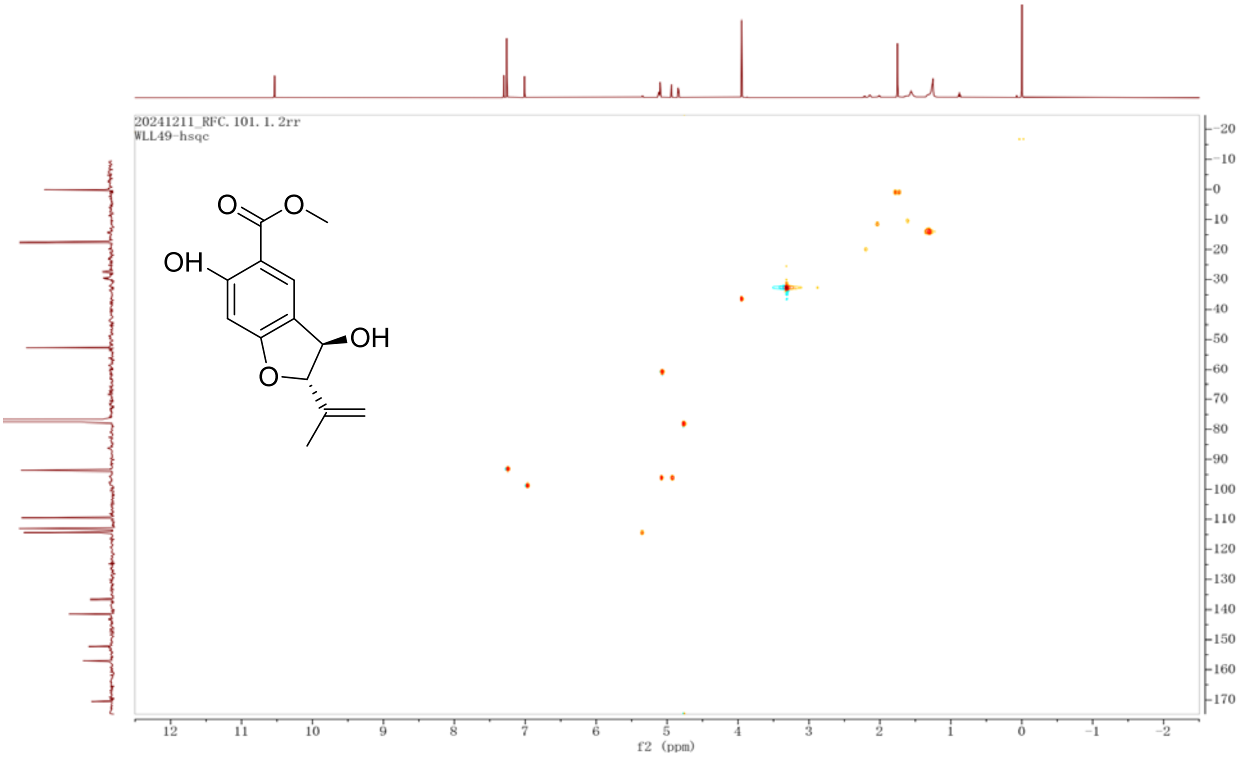

S17. HRESIMS spectrum of oberoniaensiformisin C

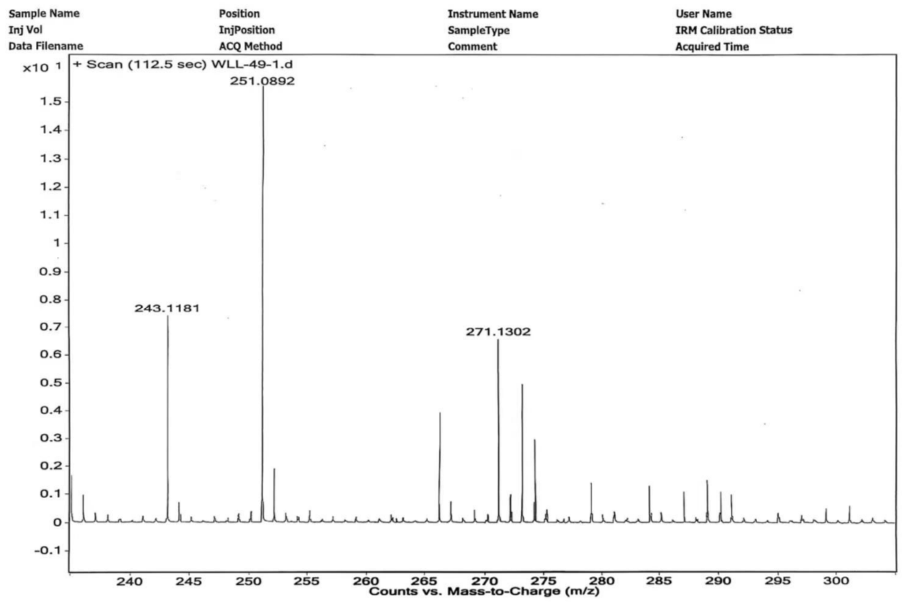

S18. IR spectrum of oberoniaensiformisin C

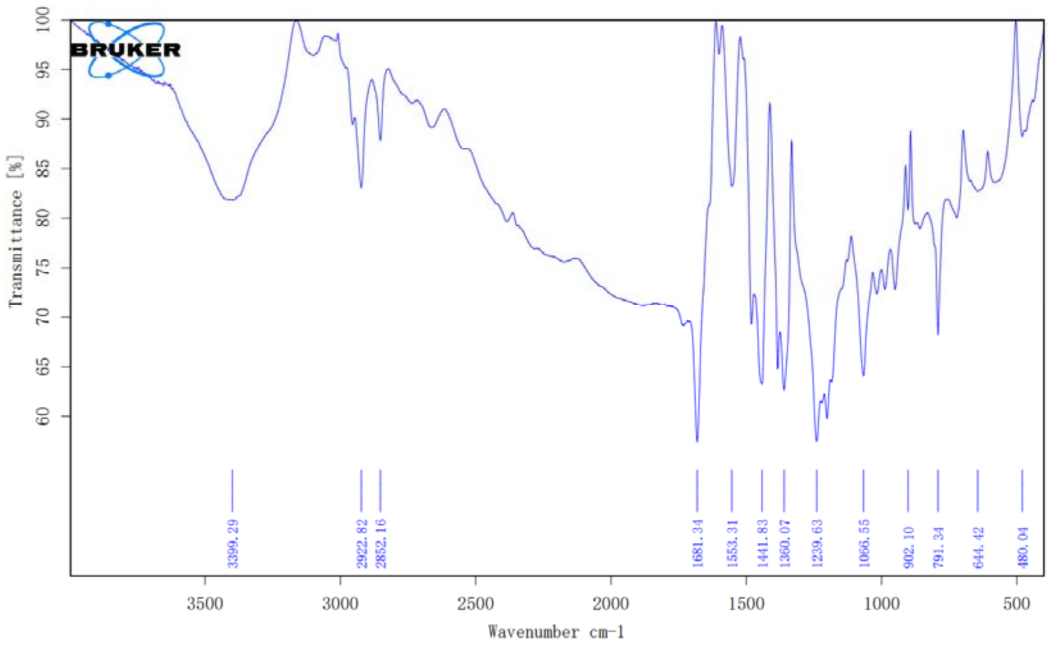

S19. ^{1}H NMR spectrum (600 MHz, CD_3OD) of oberoniaensiformisin D

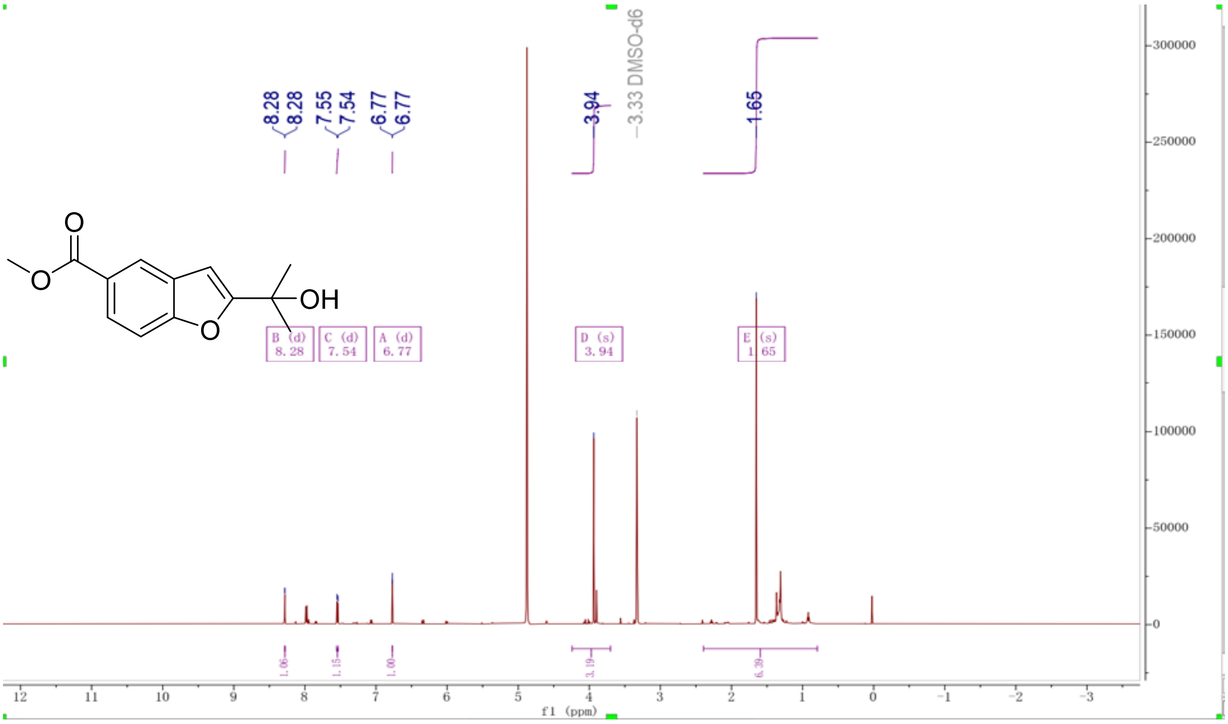

S20. ^{13}C NMR spectrum (150 MHz, CD_3OD) of oberoniaensiformisin D

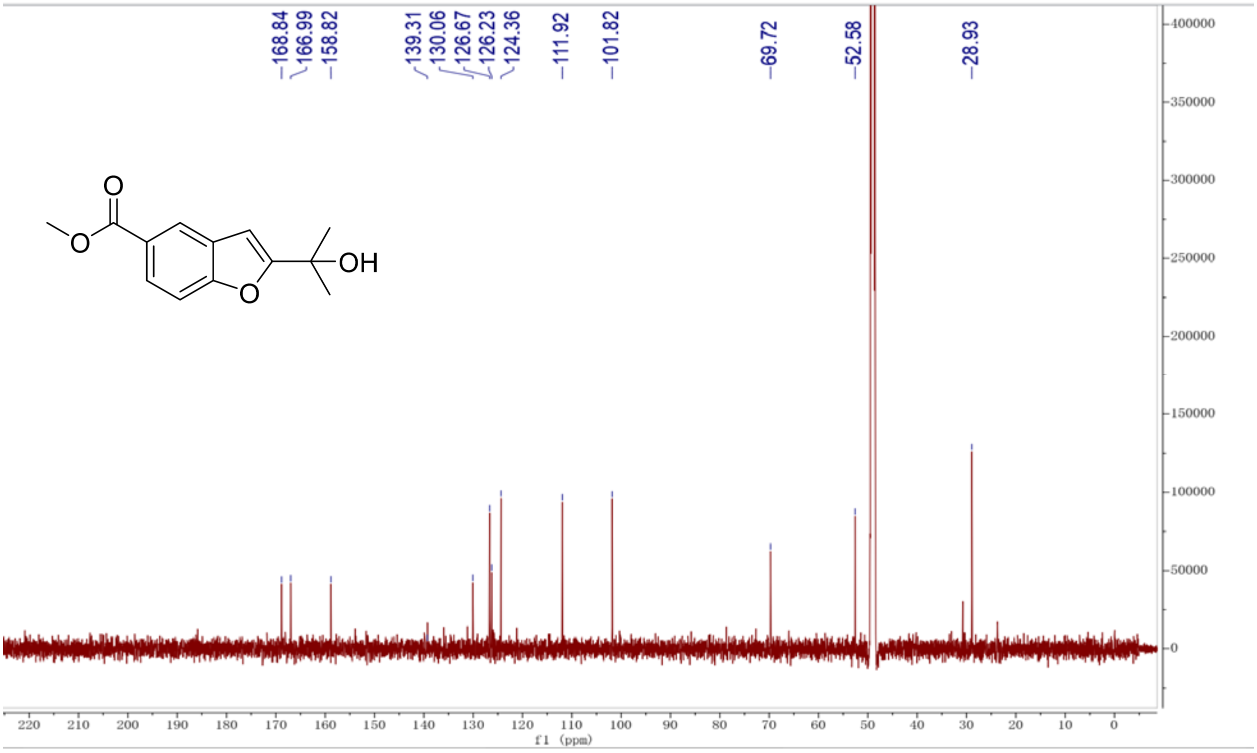

S21. HMBC spectrum (600 MHz, CD_3OD) of oberoniaensiformisin D

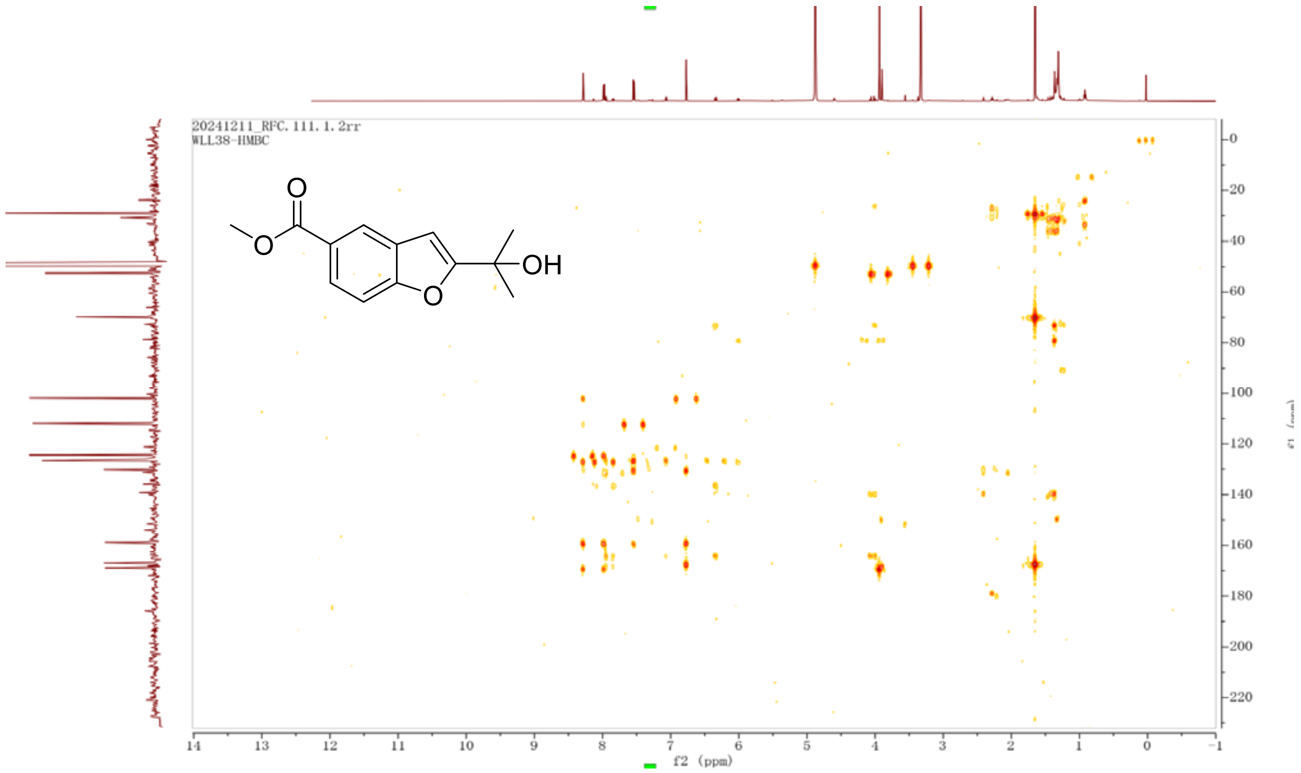

S22. HSQC spectrum (600 MHz, CD_3OD) of oberoniaensiformisin D

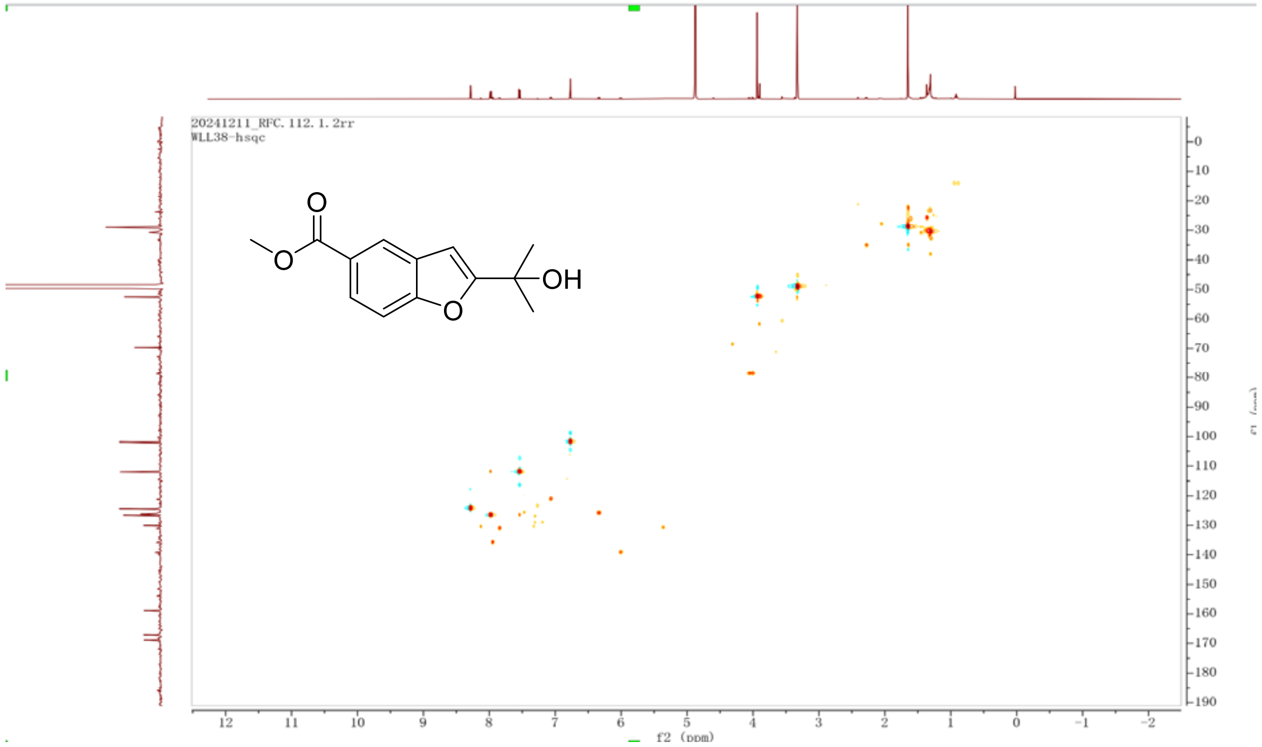

S23. HRESIMS spectrum of oberoniaensiformisin D

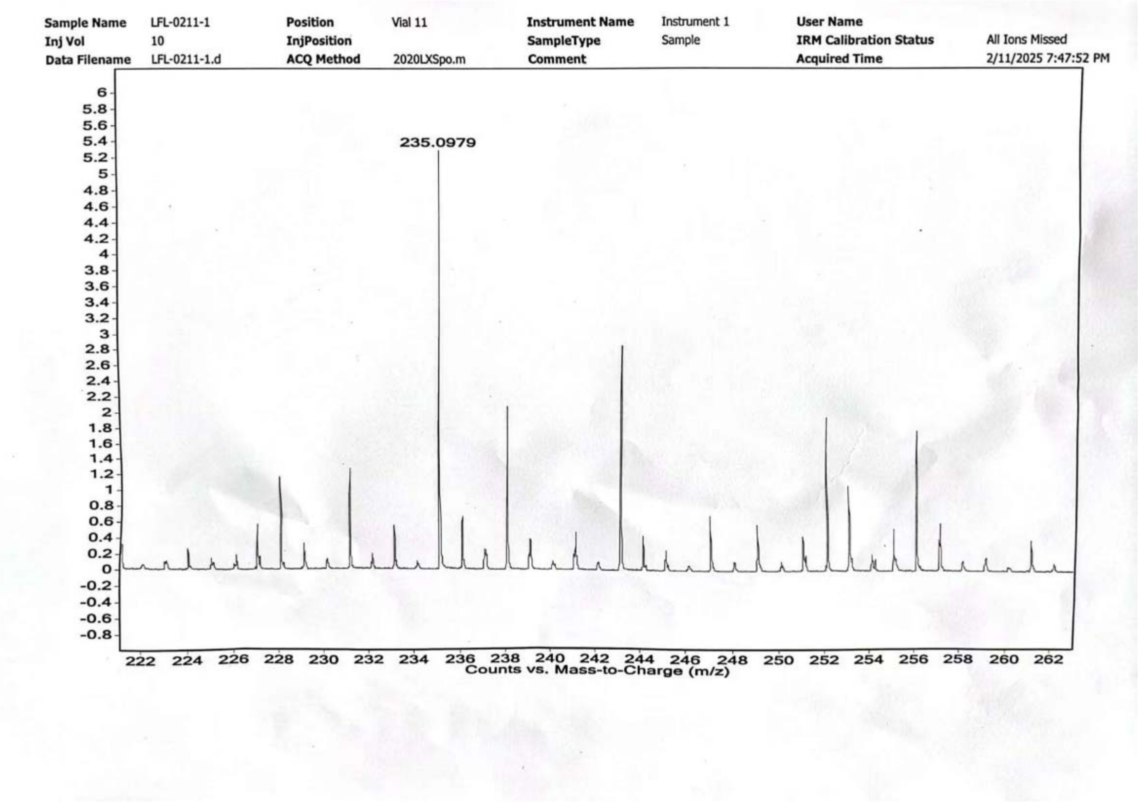

S24. IR spectrum of oberoniaensiformisin D

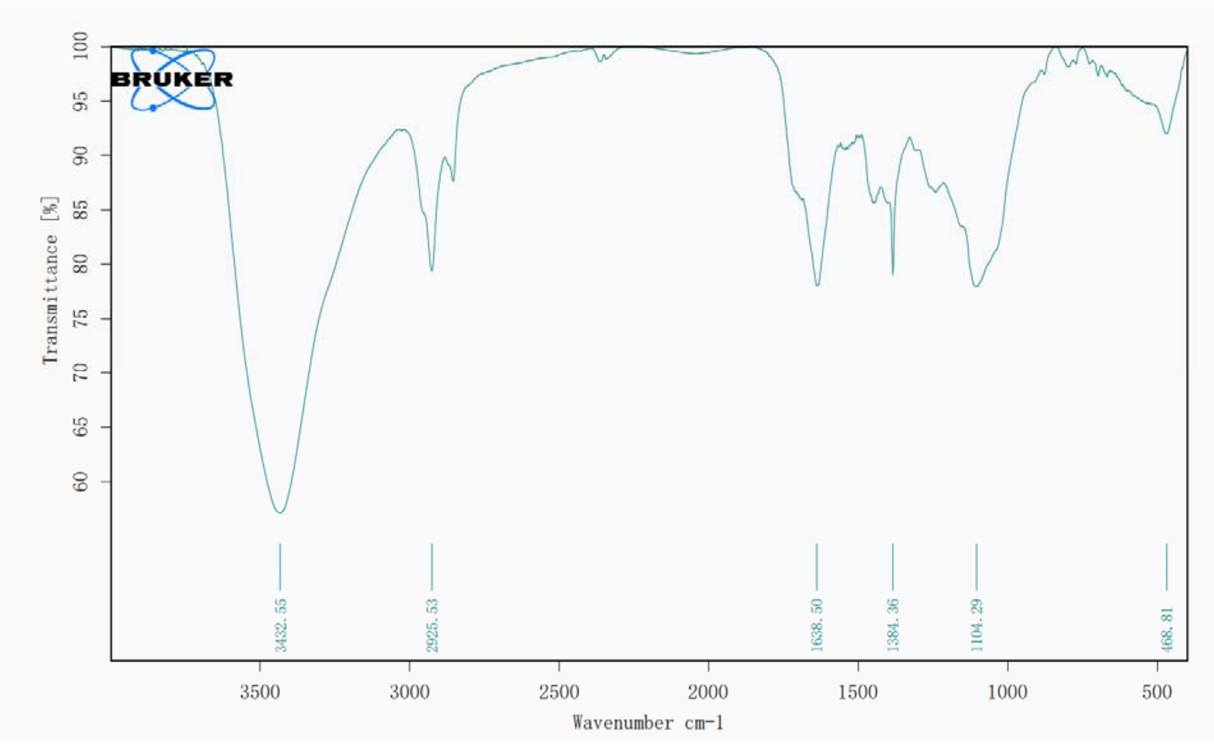

S25. ^{1}H NMR spectrum (600 MHz, DMSO-d_6) of oberoniaensiformisin E

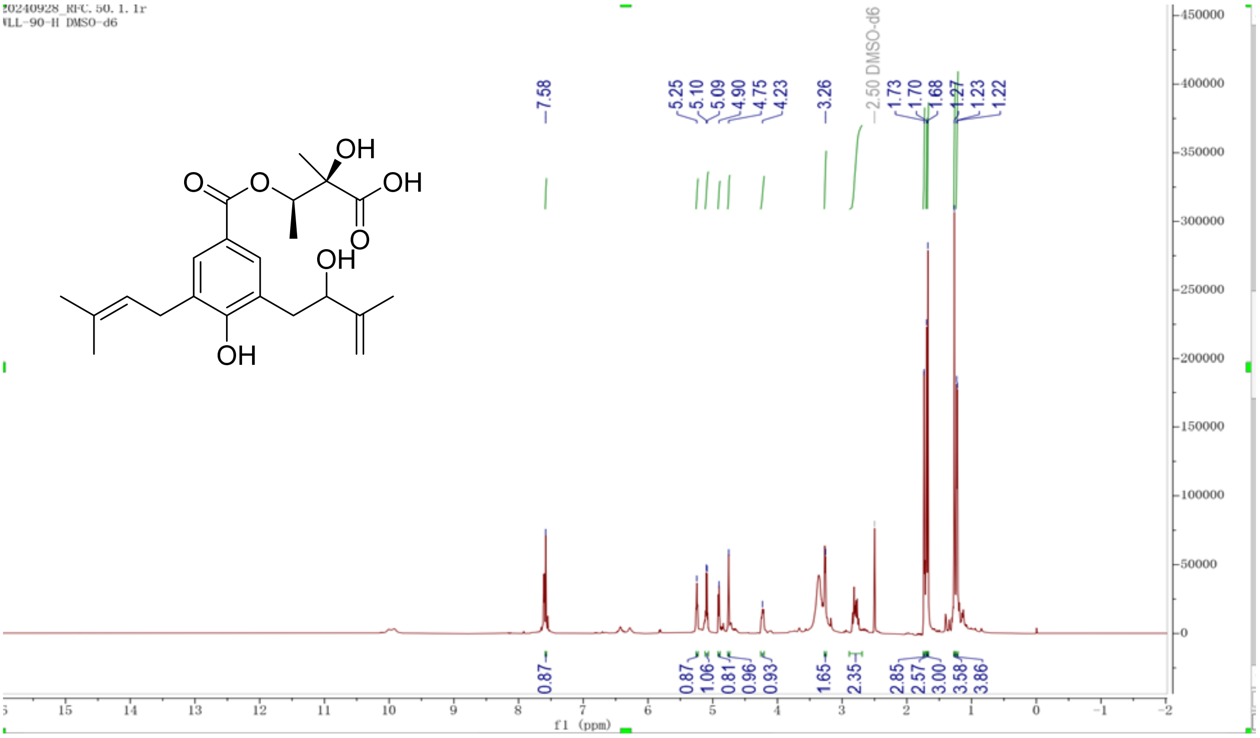

S26 ^{13}C NMR spectrum (150 MHz, DMSO-d_6) of oberoniaensiformisin E

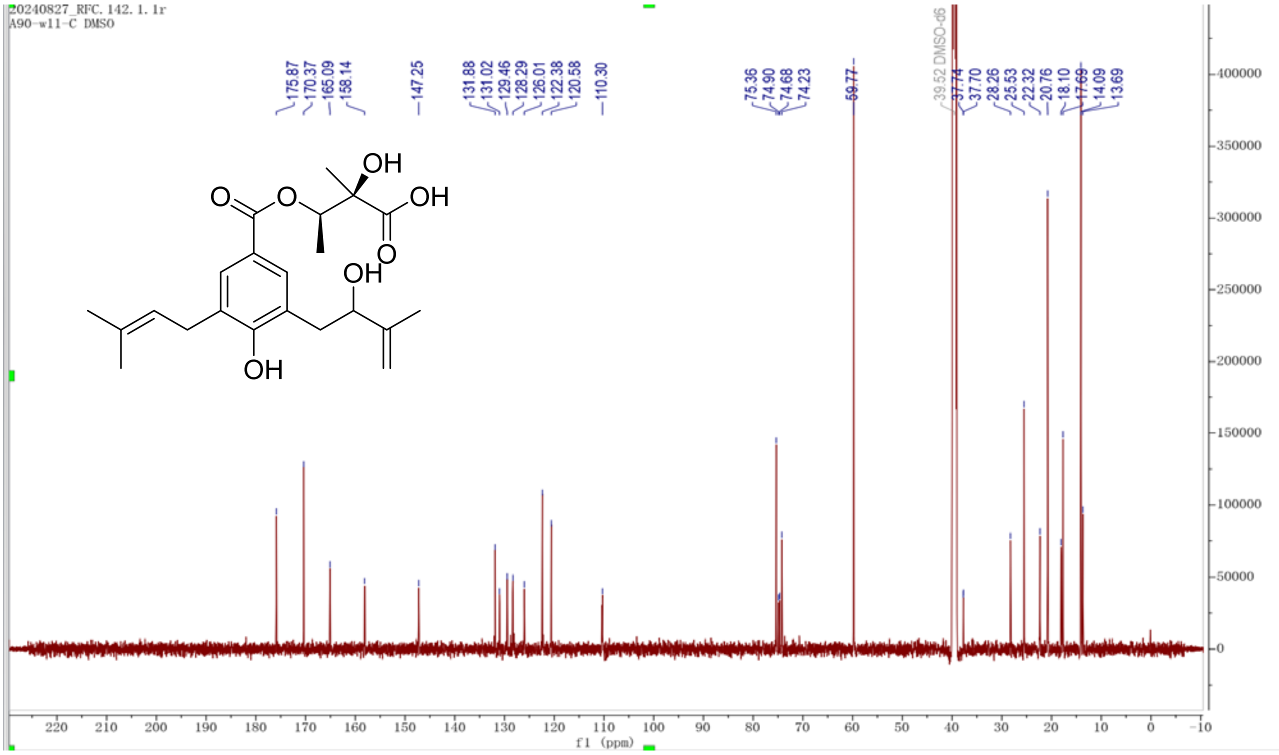

S27. HMBC spectrum (600 MHz, DMSO-d_6) of oberoniaensiformisin E (5)

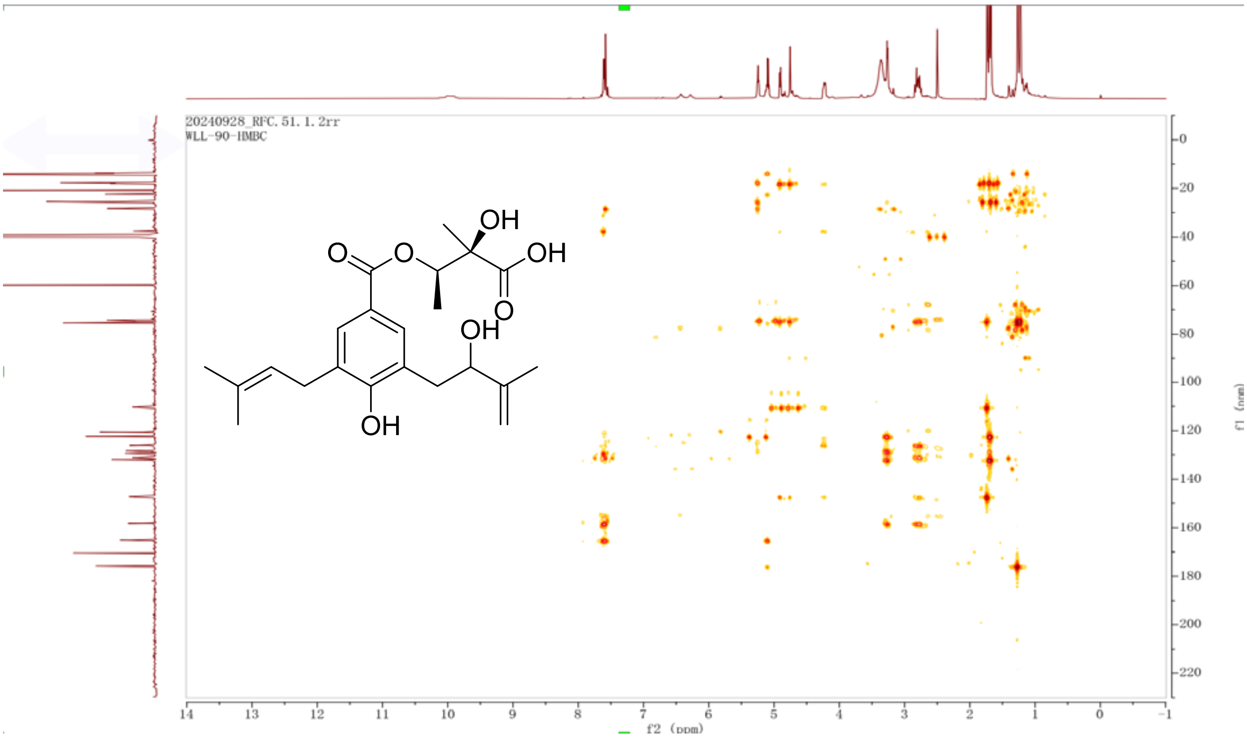

S28. HSQC spectrum (600 MHz, DMSO-d_6) of oberoniaensiformisin E

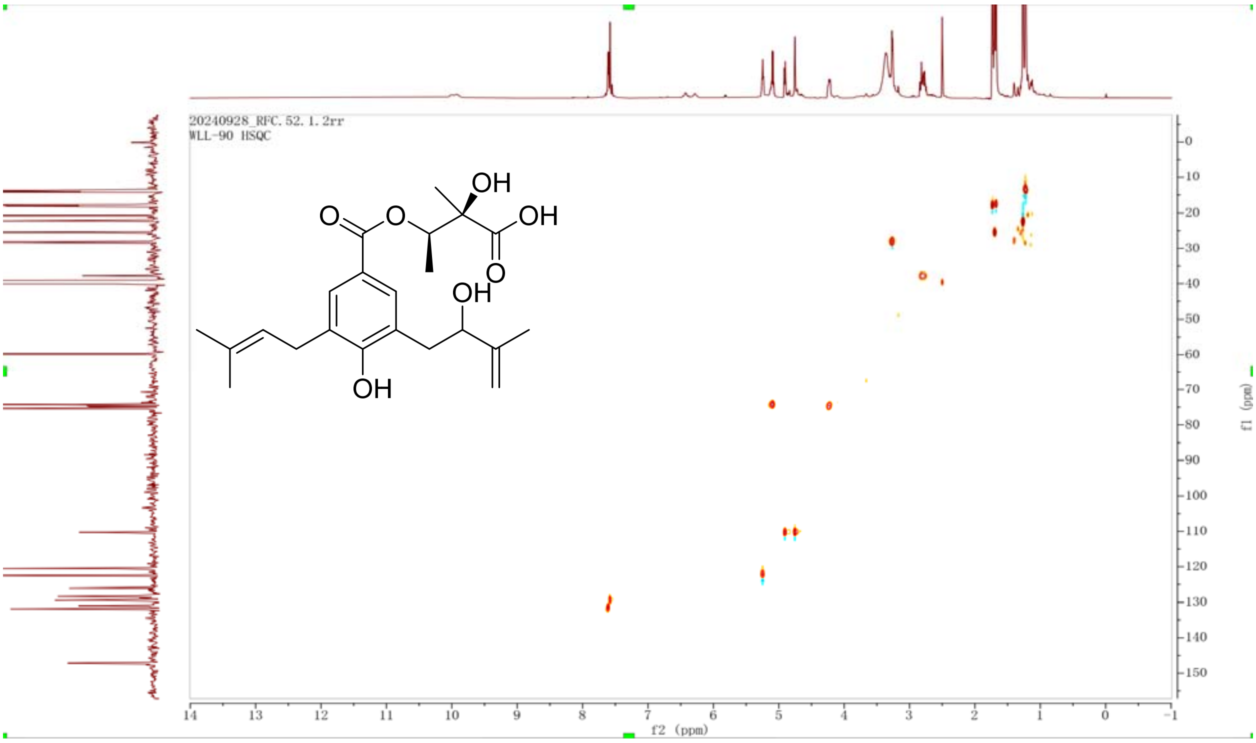

S29. COSY spectrum (600 MHz, DMSO-d_6) of oberoniaensiformisin E

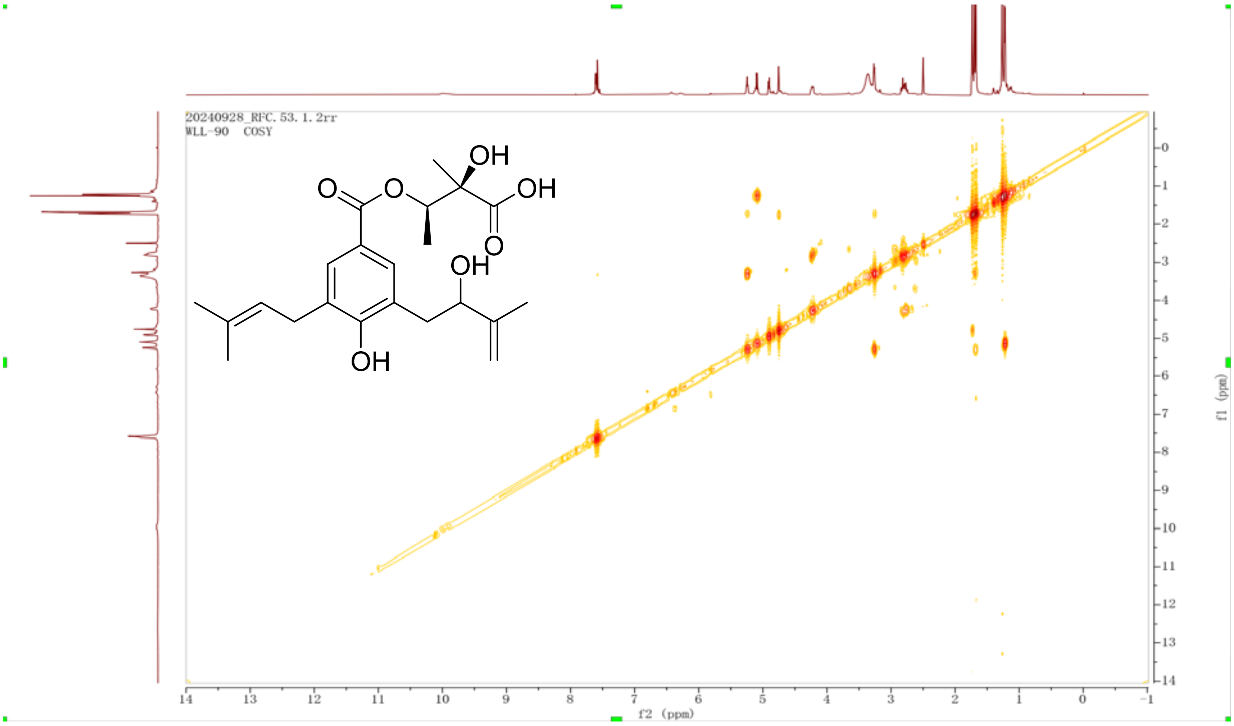

S30. HRESIMS spectrum of oberoniaensiformisin E

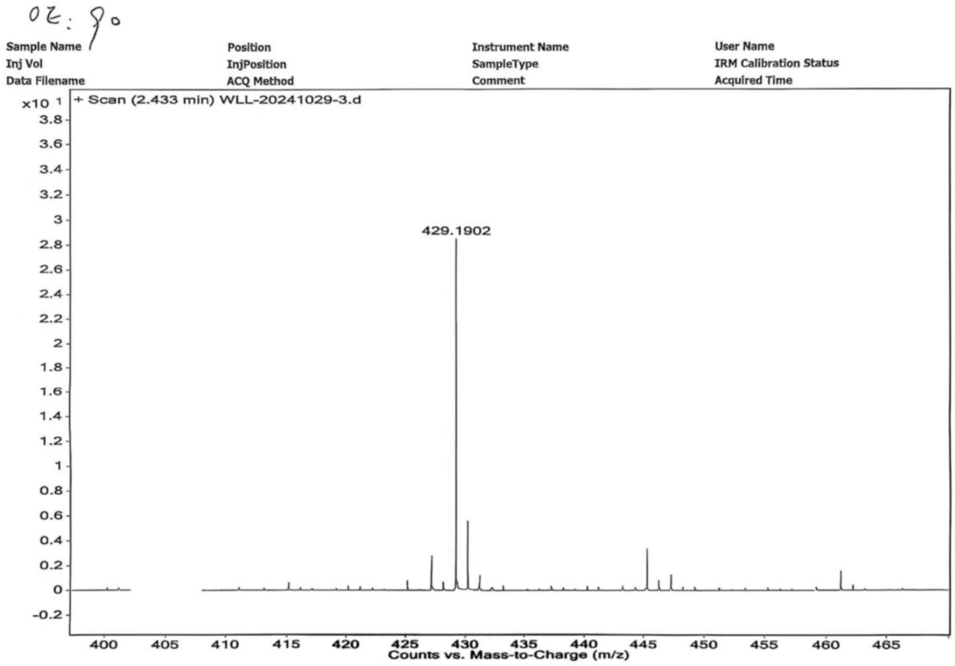

S.31. IR spectrum of oberoniaensiformisin E

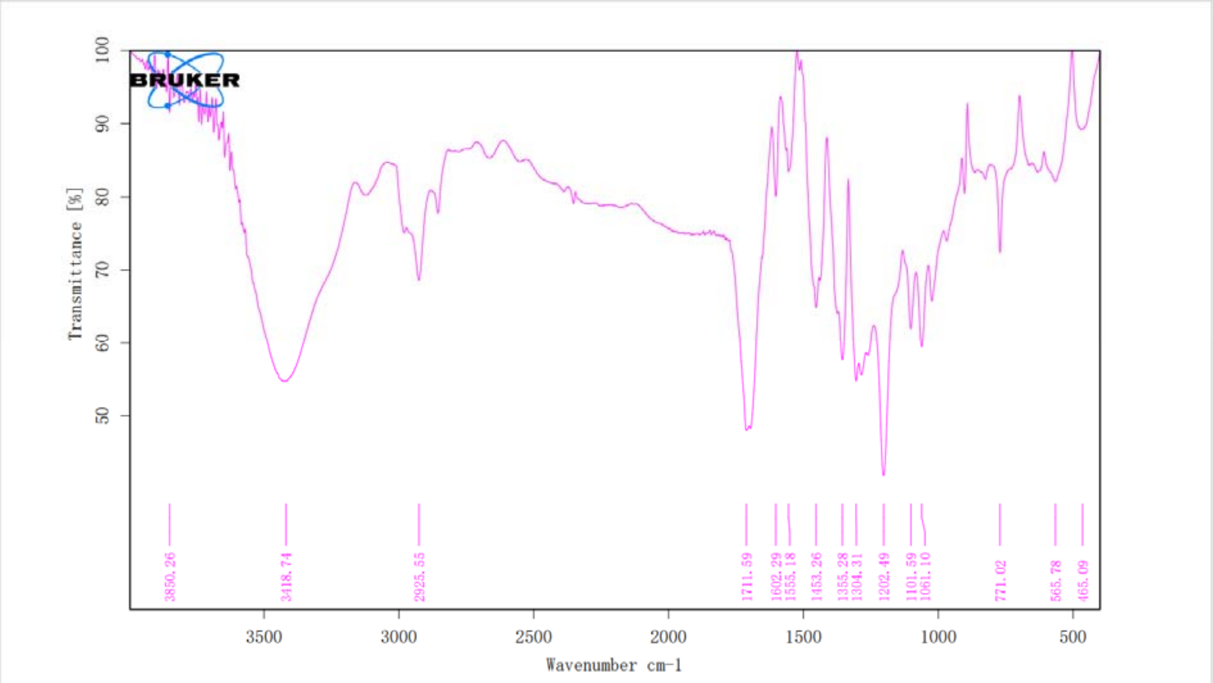

S32. HPLC chromatogram of oberoniaensiformisin E

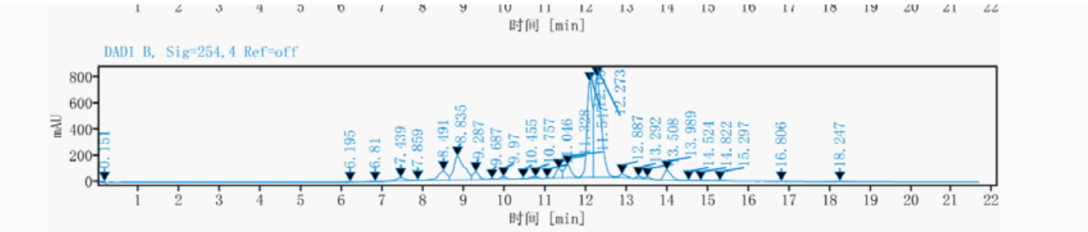

S33. ^{1}H NMR spectrum (600 MHz, DMSO-d_6) of oberoniaensiformis F

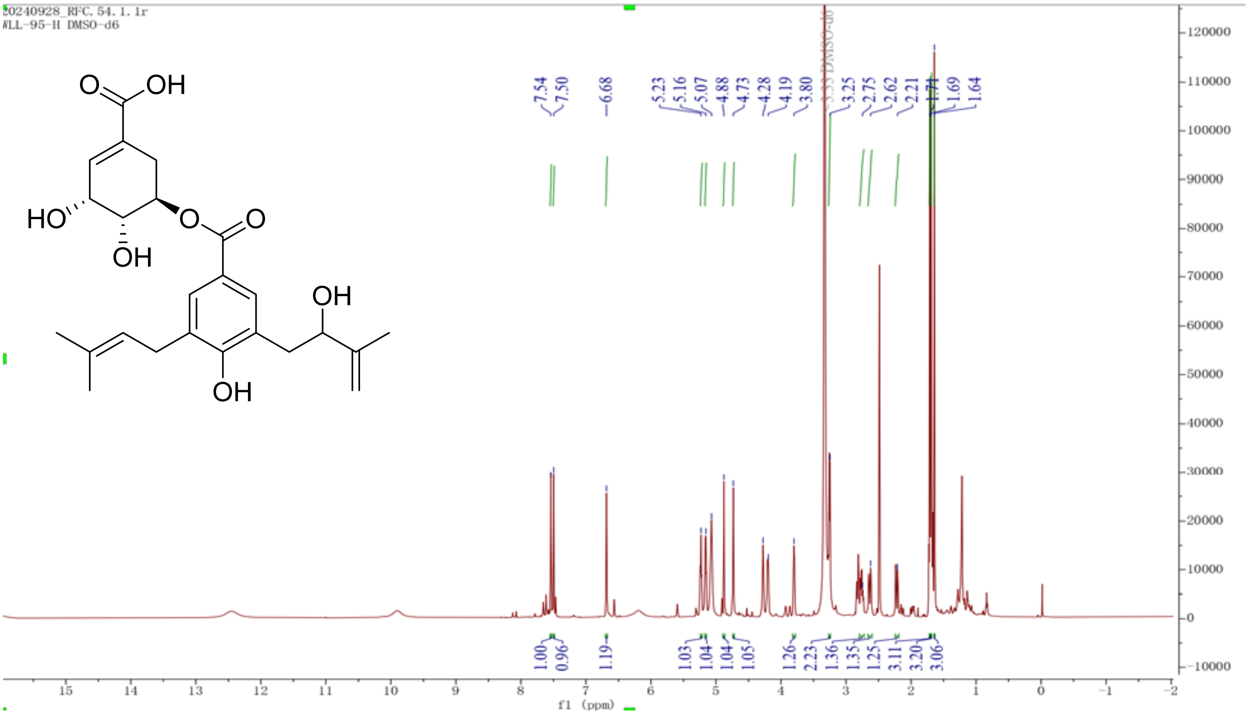

S34. ^{13}C NMR spectrum (150 MHz, DMSO-d_6) of oberoniaensiformisin F

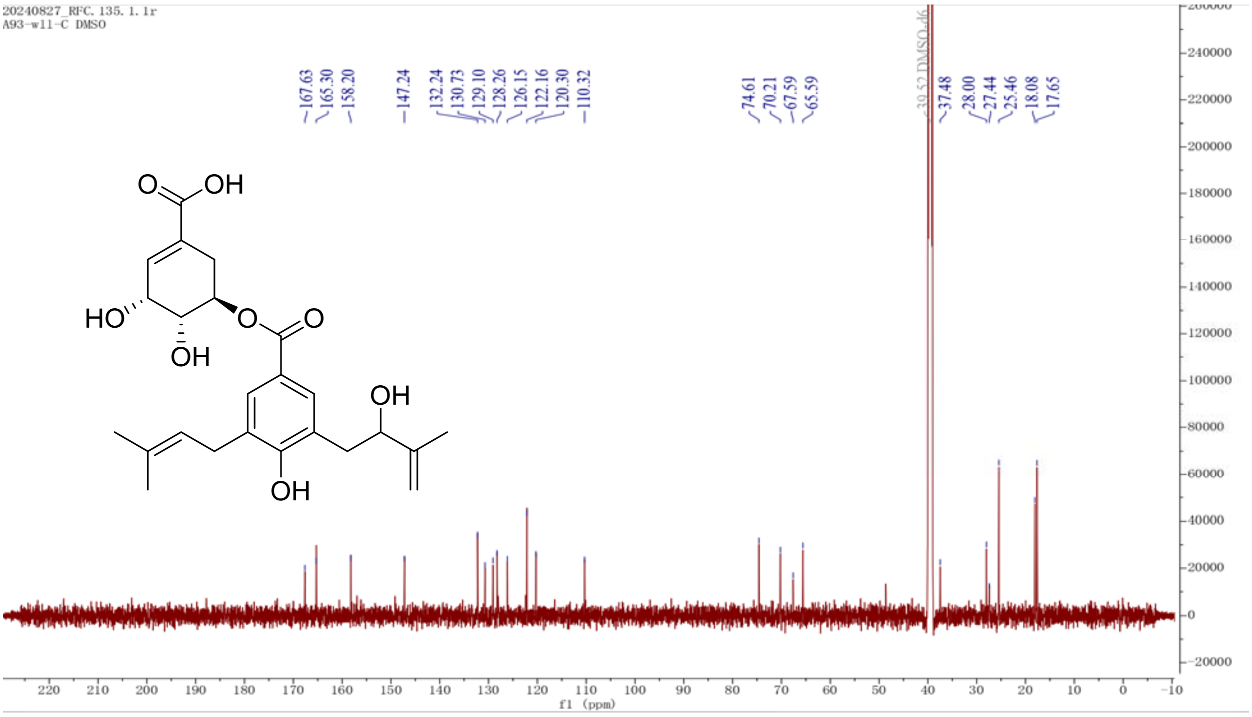

S35. HMBC spectrum (600 MHz, DMSO-d_6) of oberoniaensiformisin F

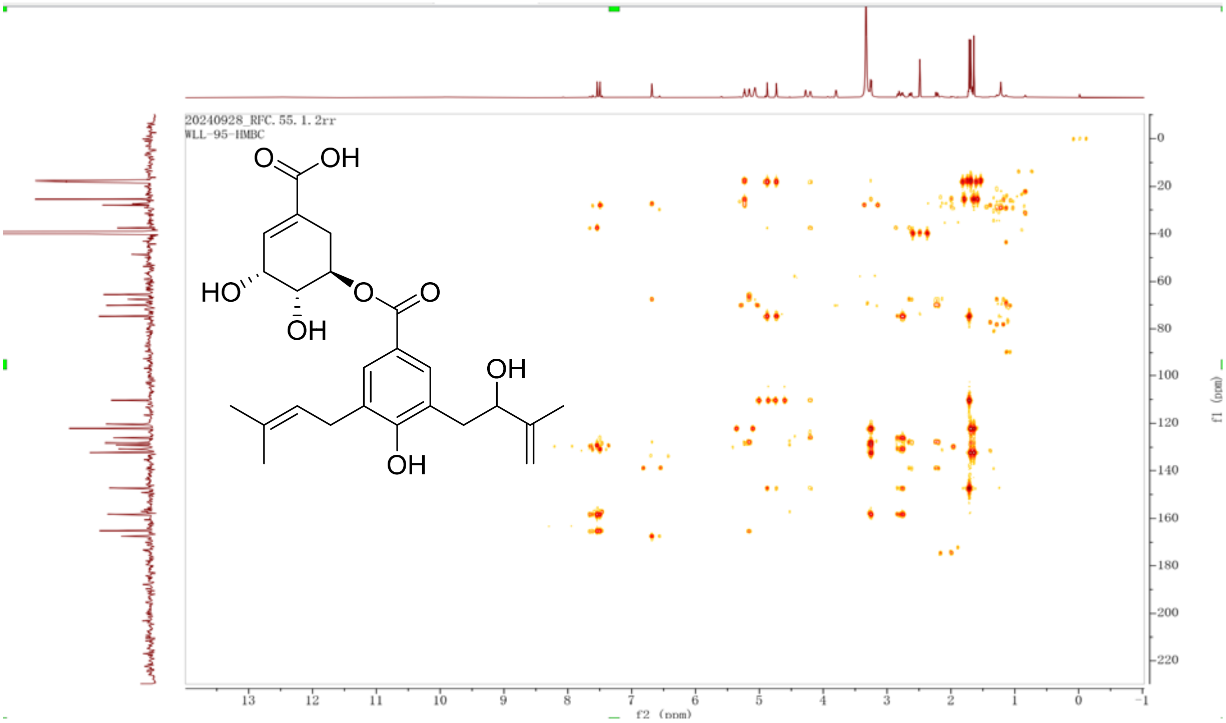

S36. HSQC spectrum (600 MHz, DMSO-d_6) of oberoniaensiformisin F

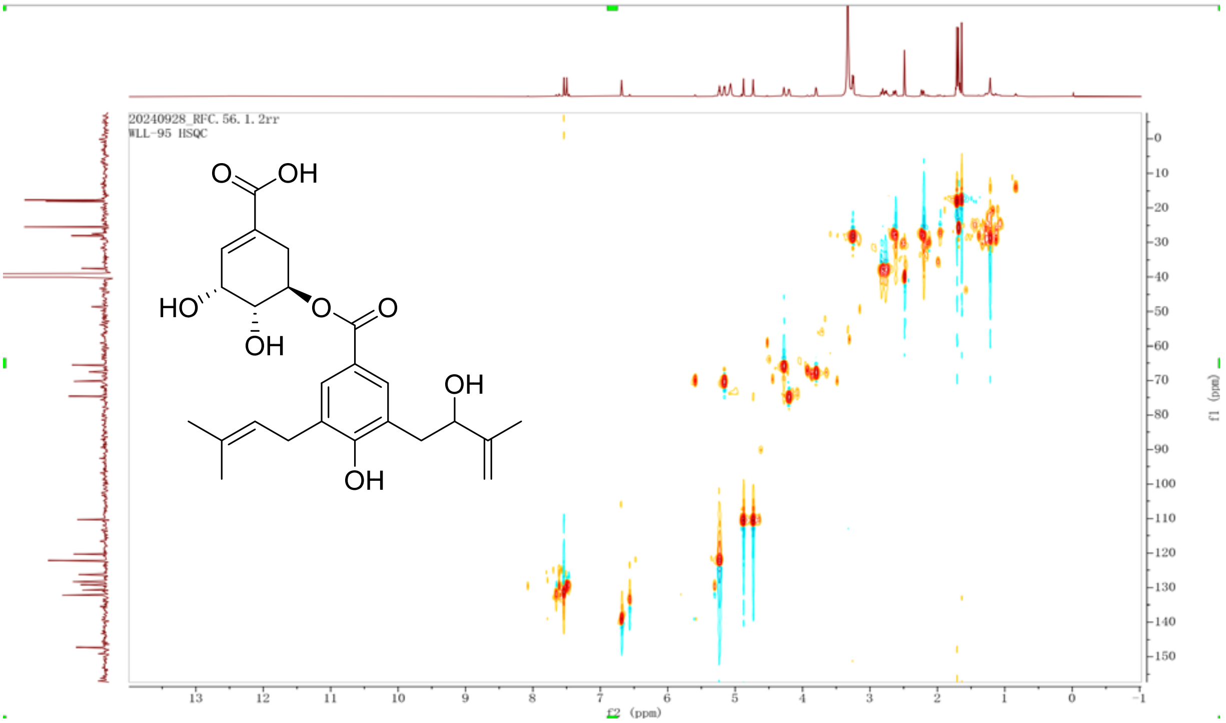

S37. COSY spectrum (600 MHz, DMSO-d_6) of oberoniaensiformisin F

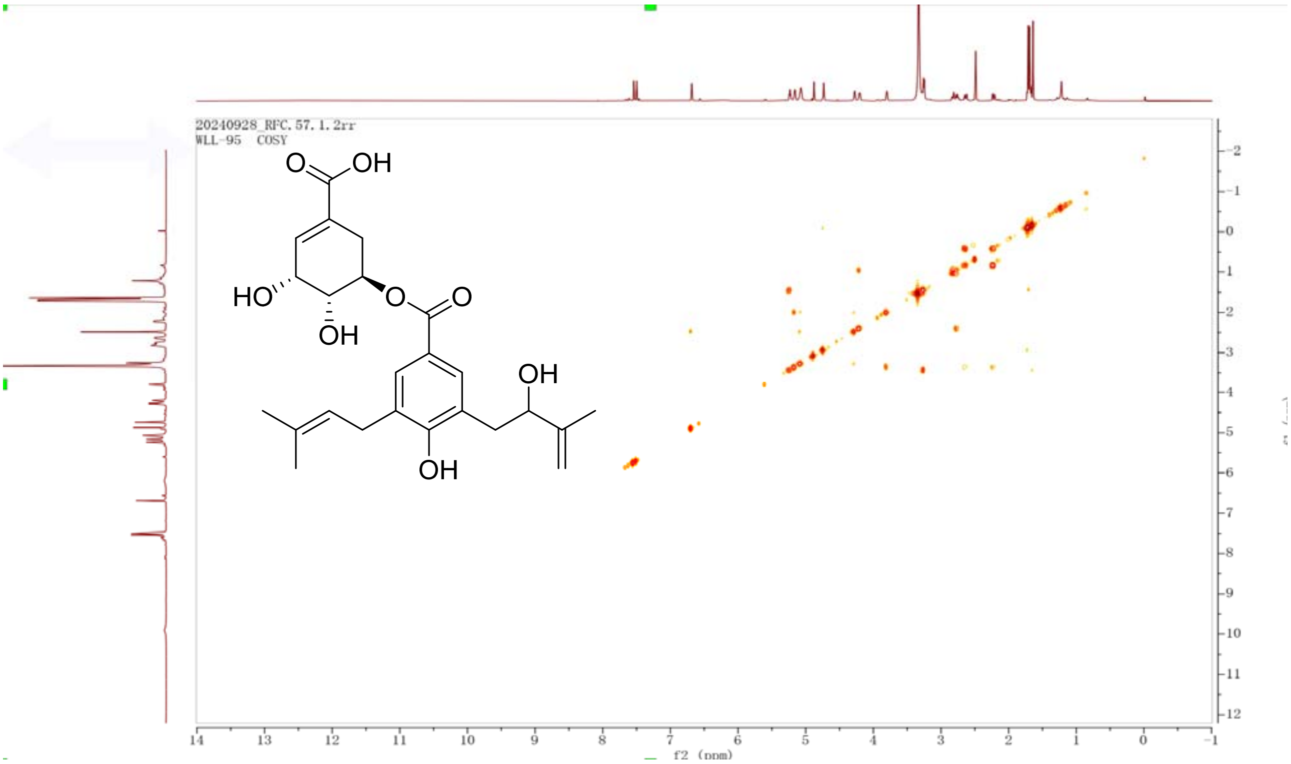

S38. HRESIMS spectrum of oberoniaensiformisin F

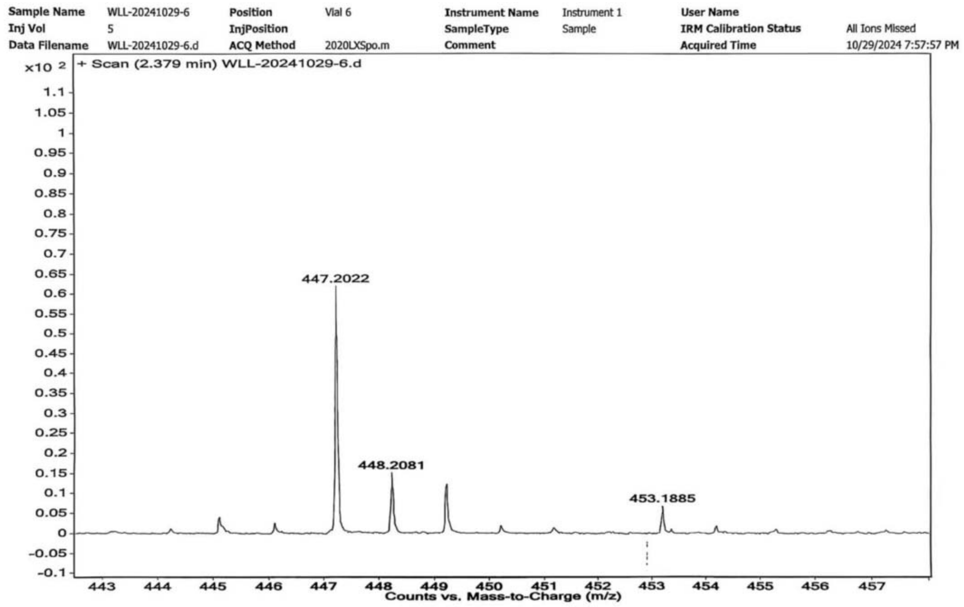

S39. IR spectrum of oberoniaensiformisin F

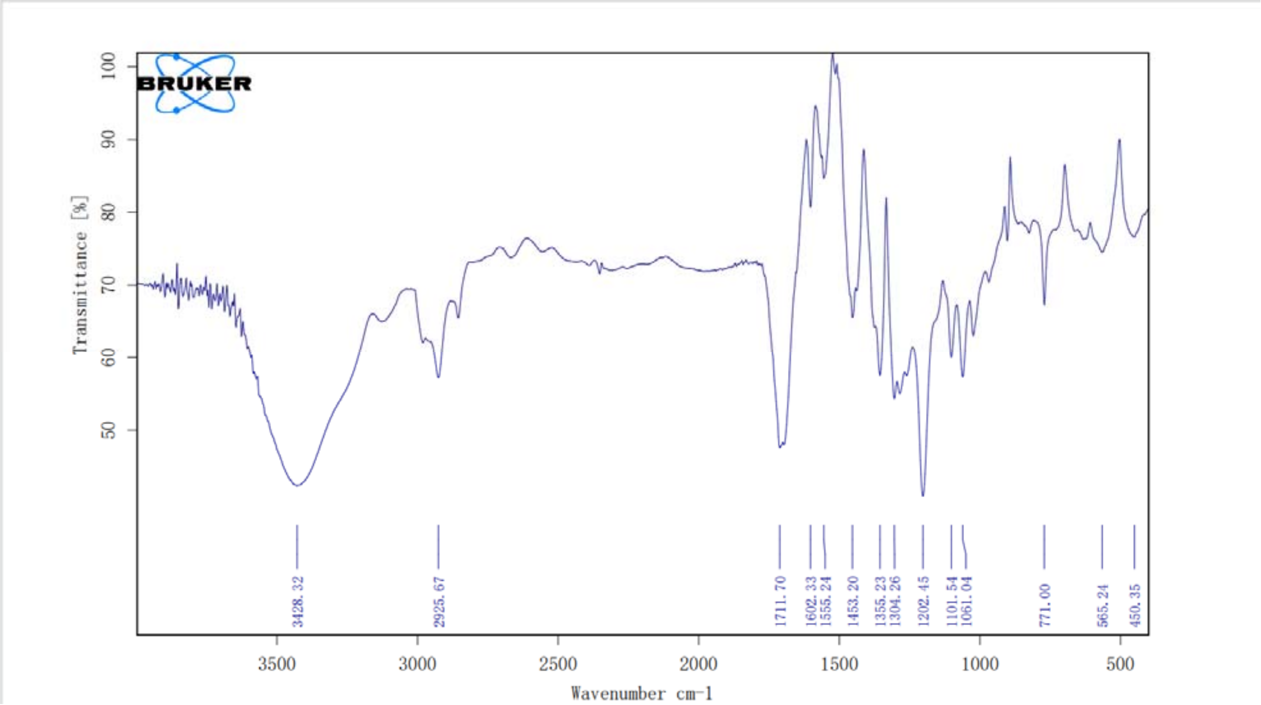

S40. HPLC chromatogram of oberoniaensiformisin F

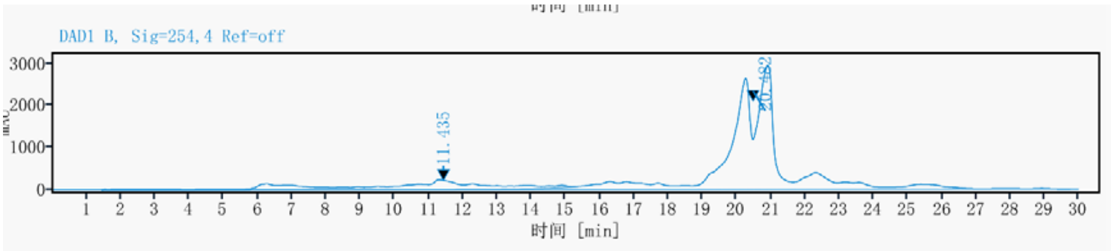

S41. ^{1}H NMR spectrum (600 MHz, CD_3OD) of oberoniaensiformisin G

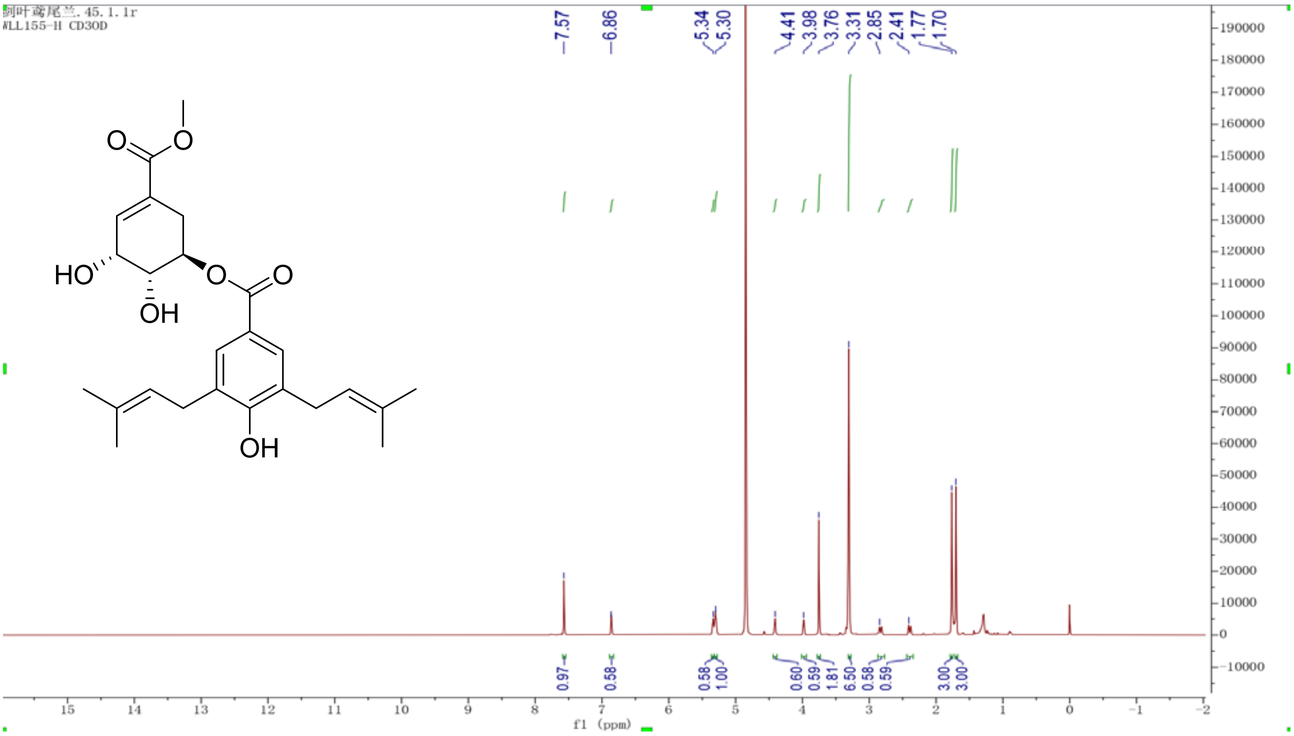

S42. ^{13}C NMR spectrum (150 MHz, CD_3OD) of oberoniaensiformisin G

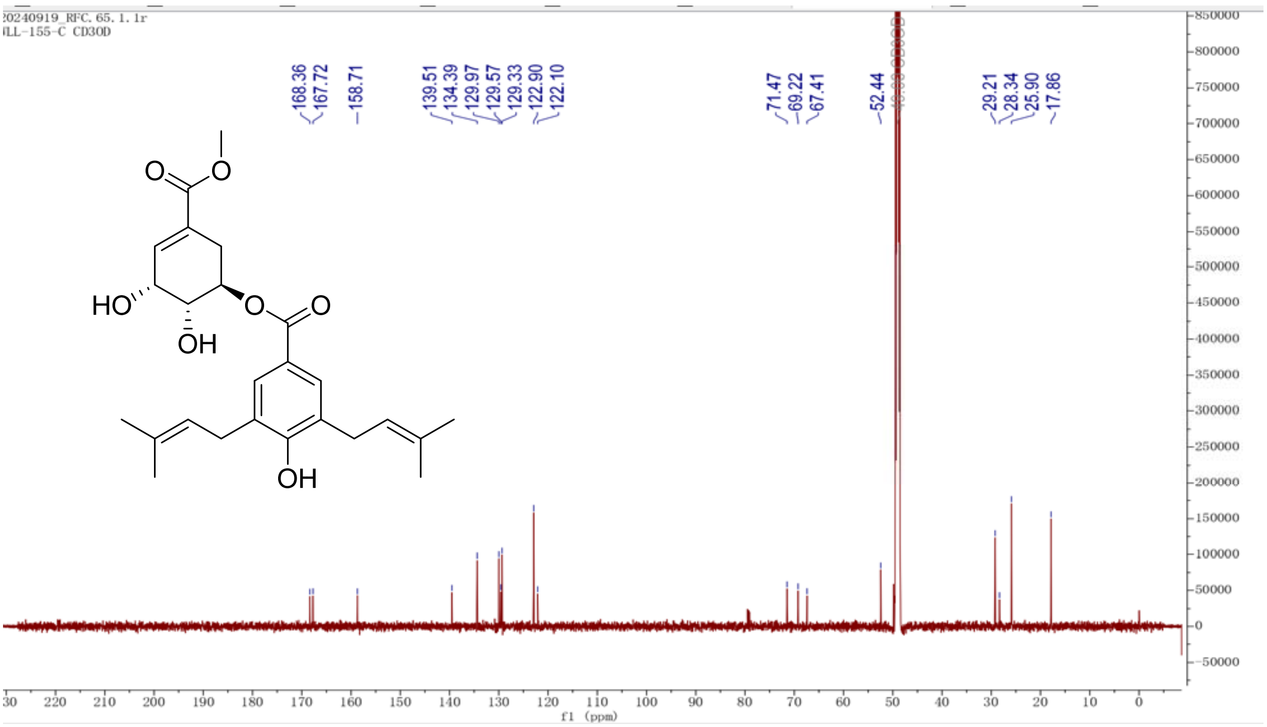

S43. HMBC spectrum (600 MHz, CD_3OD) of oberoniaensiformisin G

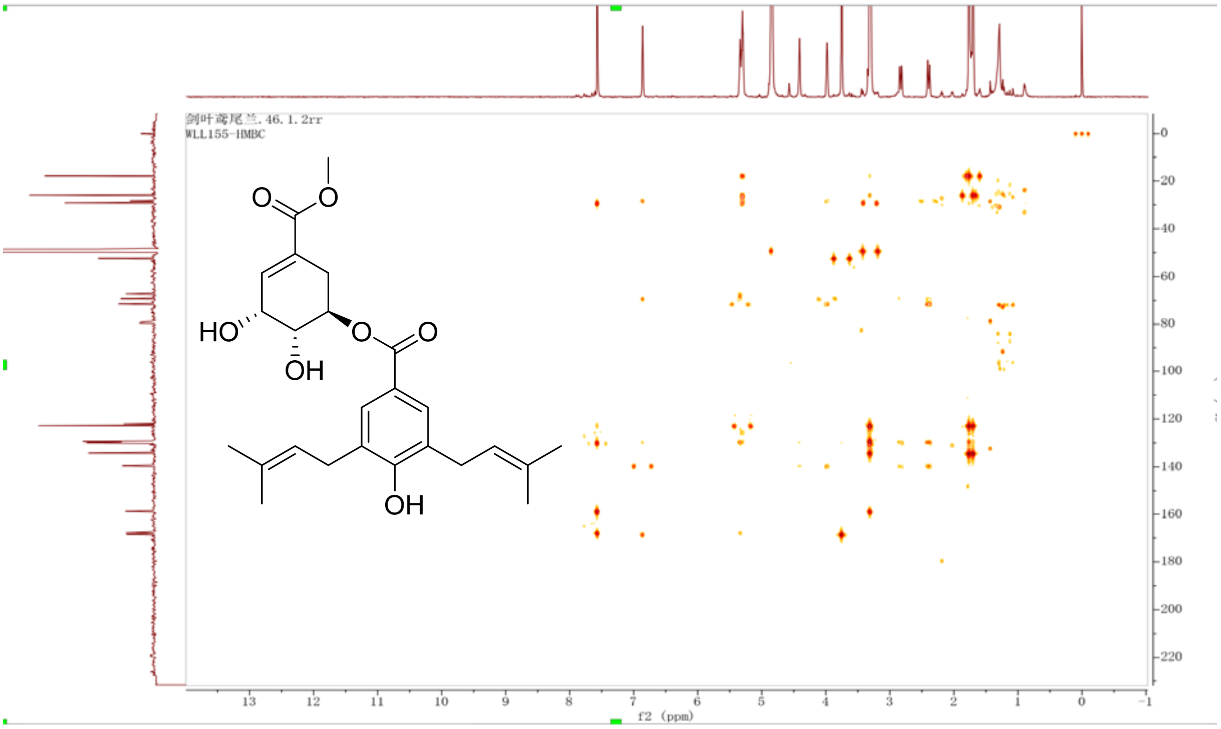

S44. HSQC spectrum (600 MHz, CD_3OD) of oberoniaensiformisin G

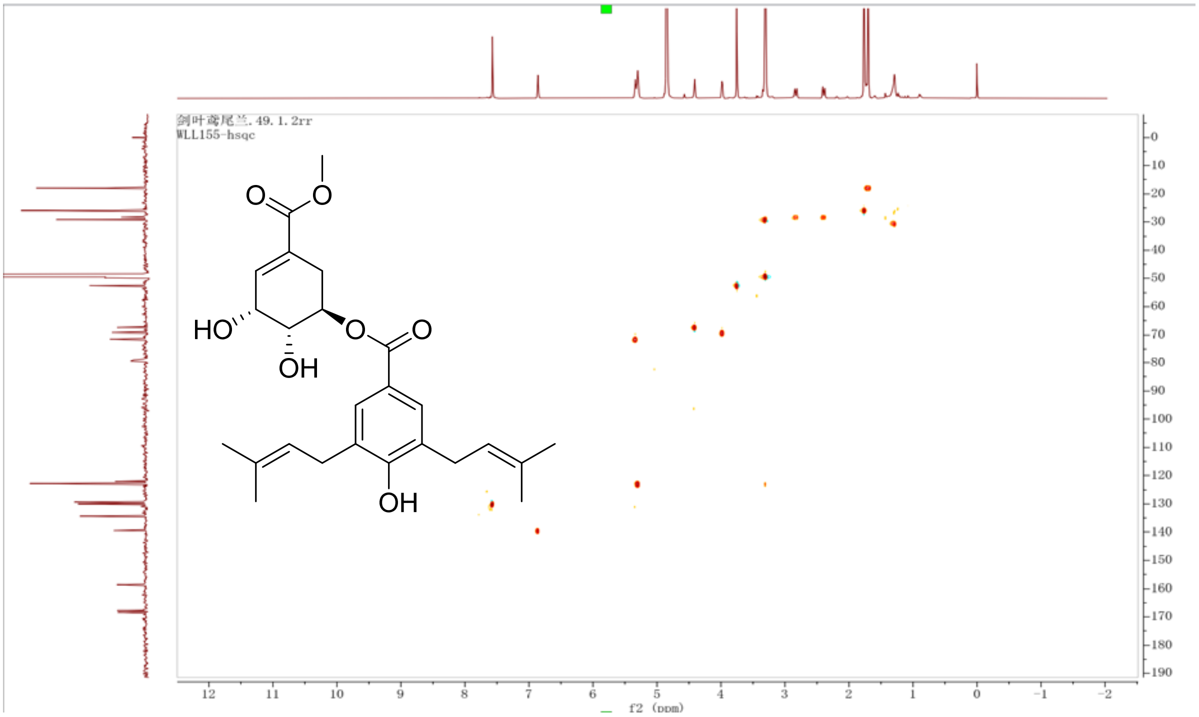

S45. COSY spectrum (600 MHz, CD_3OD) of oberoniaensiformisin G

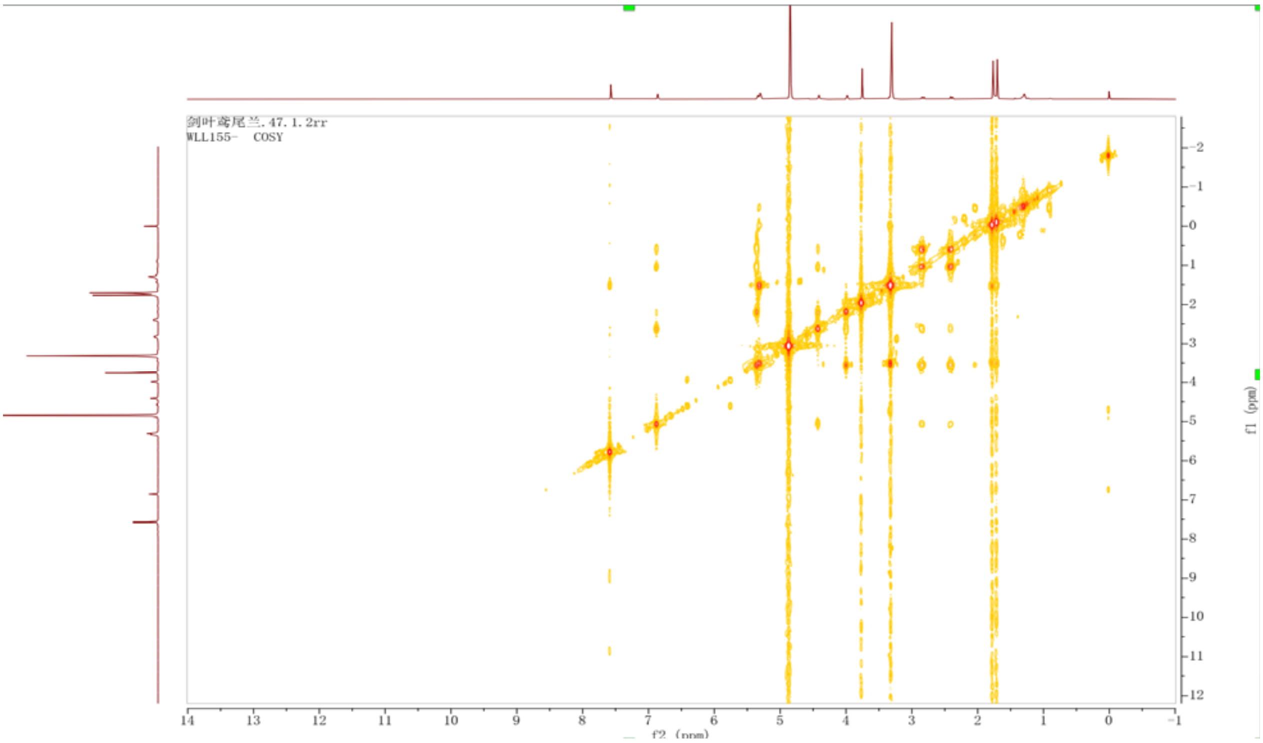

S46. NOESY spectrum (600 MHz, CD_3OD) of oberoniaensiformisin G

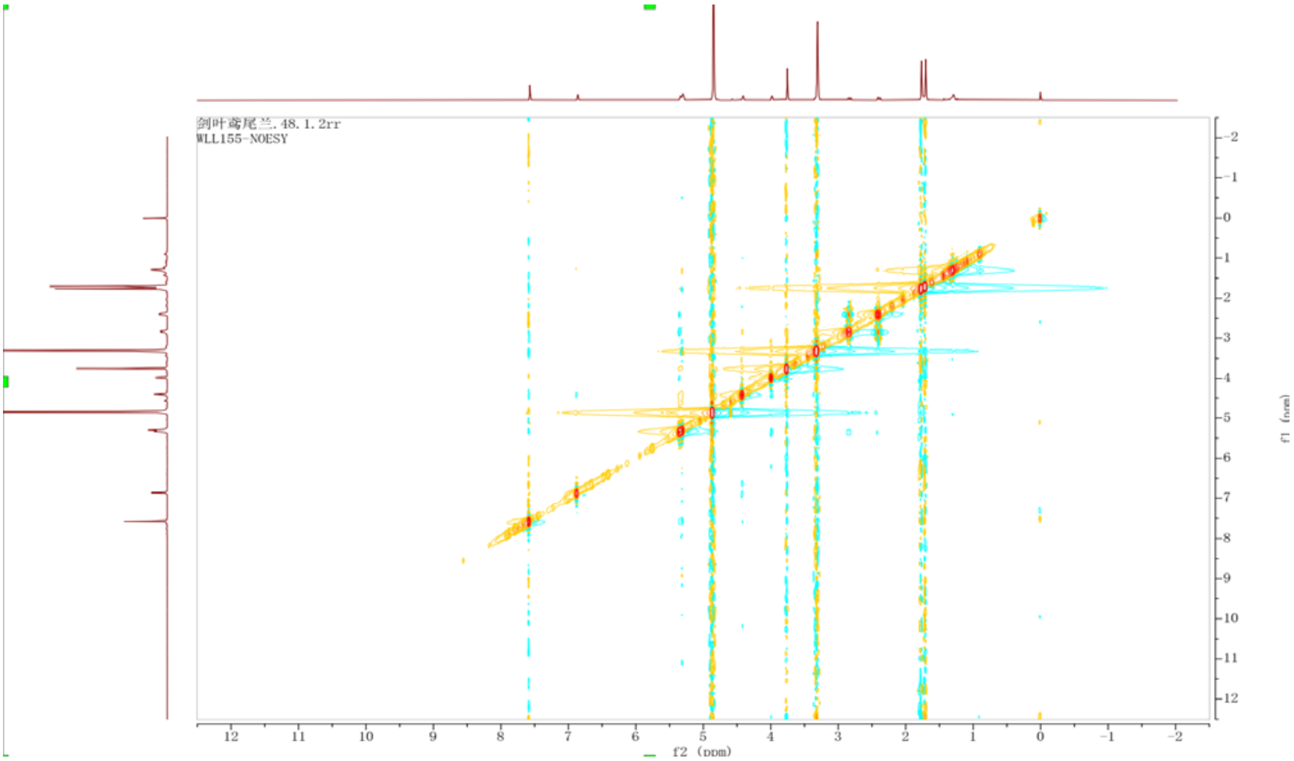

S47. HRESIMS spectrum of oberoniaensiformisin G

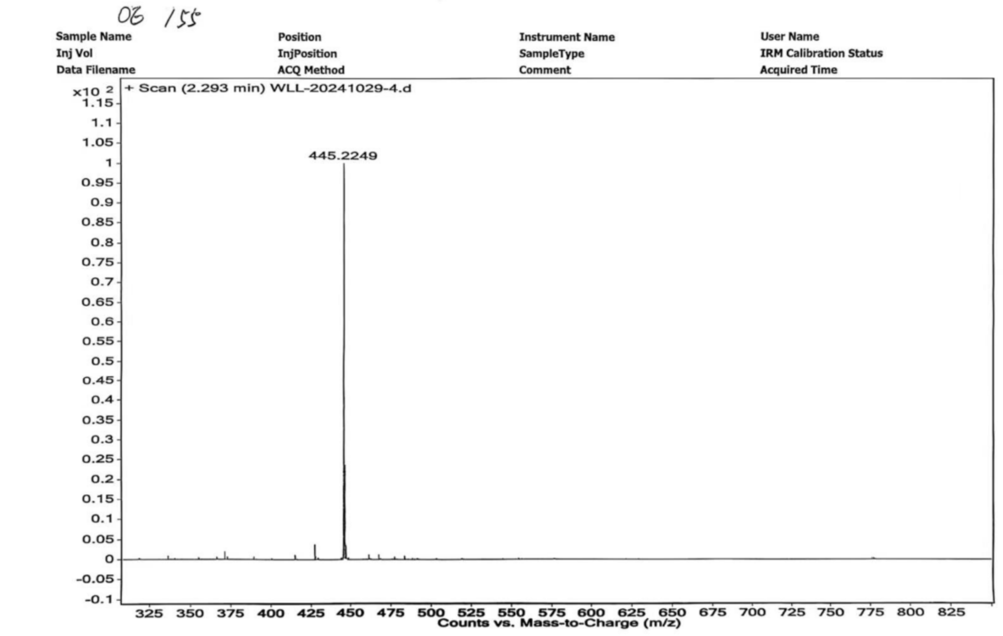

S48. IR spectrum of oberoniaensiformisin G

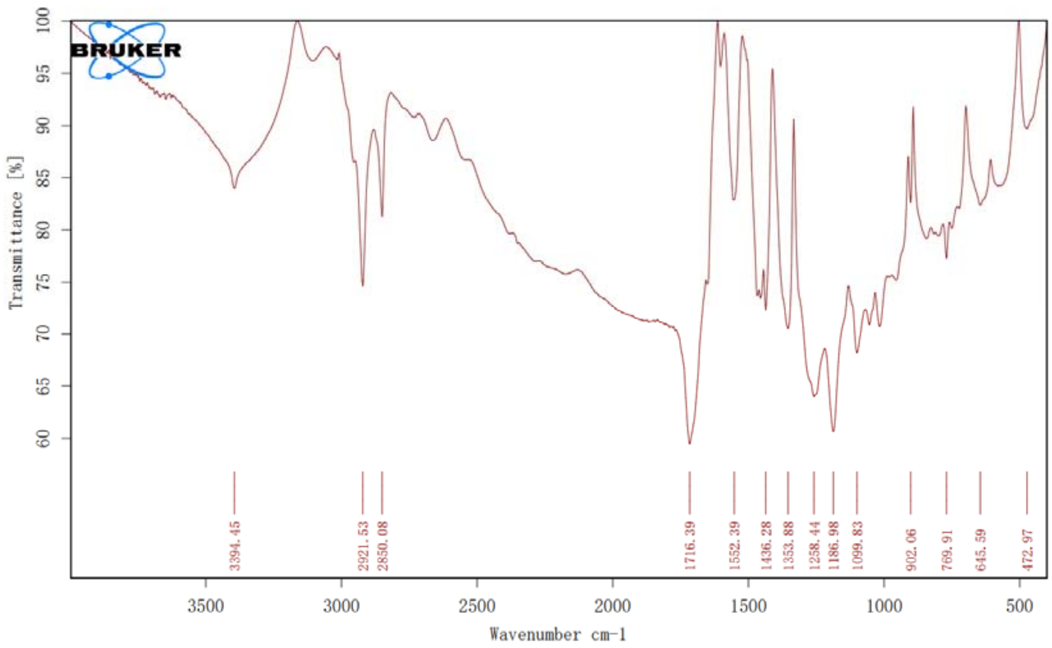

S49. Enzyme inhibition results of inactive compounds among compounds **1**–**18**.

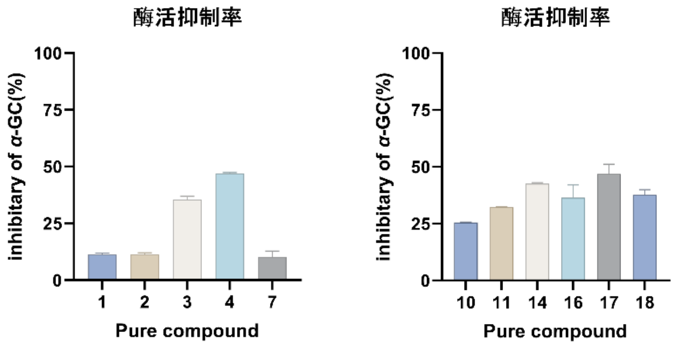

S50. Configuration identification of compound **3**:

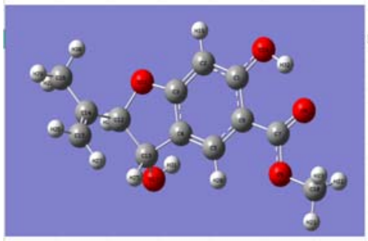

Experimental value	Conformation	3	4	1	6	7	8	
	Weight (%)	40. 82	38. 7628	11. 1011	6. 62304	1. 3847	1. 30837	
	Calculation ID	0. 4082	0. 387628	0. 111011	0. 06623	0. 013847	0. 013084	Weighted
156. 80	C1	14. 9752	14. 9321	15. 3848	15. 2202	15. 3168	14. 9134	15. 02411
52. 46	C10	133. 6861	133. 6503	133. 788	133. 8092	133. 7346	133. 6949	133. 6925
93. 49	C12	88. 0034	84. 9335	86. 3203	88. 1919	89. 2874	88. 1688	86. 65902
76. 75	C13	106. 5873	106. 9378	107. 6065	106. 6265	109. 0186	112. 6289	106. 9516
141. 28	C14	36. 4256	34. 3727	34. 0859	36. 9515	35. 752	36. 5456	35. 39718
112. 86	C15	74. 0011	66. 8085	67. 1633	73. 4329	71. 5891	70. 6147	70. 33865
17. 48	C16	166. 937	170. 338	169. 5954	166. 9121	164. 4981	163. 2555	168. 4669
109. 31	C2	86. 3596	86. 1655	86. 339	86. 619	86. 1343	85. 8665	86. 28969
152. 14	C3	14. 8381	14. 6787	15. 3795	14. 8583	16. 9282	16. 1291	14. 88359
113. 09	C4	64. 458	63. 3735	64. 1913	65. 1637	63. 0638	63. 5613	64. 02372
114. 12	C5	52. 4825	52. 9139	53. 7864	52. 5619	54. 3911	52. 7165	52. 82922
136. 45	C6	78. 774	78. 9453	79. 2555	79. 018	79. 298	79. 0093	78. 92035
170. 35	C7	10. 8173	10. 8145	10. 6874	10. 7044	10. 7093	10. 8136	10. 79277
	H19	25. 236	25. 2771	25. 3118	25. 2529	25. 333	25. 3162	25. 26386
	H20	23. 5863	23. 5761	23. 6552	23. 6008	23. 6317	23. 528	23. 59082
	H21	27. 9451	27. 9706	27. 9851	27. 9446	27. 9736	27. 975	27. 96018
	H22	27. 982	27. 9334	27. 9386	27. 9458	27. 9301	27. 9434	27. 95472
	H23	27. 9441	27. 9321	27. 9403	27. 9921	27. 9336	27. 9415	27. 94203
	H24	26. 8708	26. 8345	26. 8666	26. 8067	26. 9145	26. 7945	26. 85163
	H25	26. 9709	26. 9288	26. 5328	26. 6887	26. 4043	26. 5208	26. 87352
	H26	26. 6596	26. 4952	26. 4563	26. 6258	26. 4553	26. 5117	26. 56631
	H27	26. 5722	26. 3702	26. 3979	26. 4465	26. 3982	26. 6682	26. 46507
	H28	29. 8587	30. 1191	30. 282	30. 0018	29. 8571	29. 8808	30. 01638
	H29	29. 8547	30. 1847	29. 7919	29. 8049	29. 8573	29. 9503	29. 97364
	H30	29. 9316	30. 5506	30. 047	29. 9817	29. 4652	29. 4412	30. 1748
	H31	30. 6035	30. 6995	29. 8899	29. 8448	30. 8446	30. 831	30. 51756
	H32	20. 1741	20. 1557	20. 2101	20. 2031	20. 2212	20. 1894	20. 17374

Experime	Conformation	1	3	4	
	Weight (%)	74. 4215	17. 0954	8. 4831	
	Calculation ID	0. 744215	0. 170954	0. 084831	Weighted
156. 80	C1	9. 4374	9. 4125	9. 2083	9. 413708463
52. 46	C10	129. 162	129. 2206	129. 0654	129. 1638232
93. 49	C12	87. 2659	85. 3492	83. 7792	86. 64245222
76. 75	C13	106. 3433	103. 7686	102. 3812	105. 5670358
141. 28	C14	31. 8814	28. 591	29. 9812	31. 15769709
112. 86	C15	68. 432	65. 2624	63. 0251	67. 43147147
17. 48	C16	160. 6064	161. 3512	162. 4167	160. 8872961
109. 31	C2	82. 013	82. 1528	81. 6282	82. 0042564
152. 14	C3	10. 8571	9. 2492	9. 7261	10. 4862792
113. 09	C4	57. 8698	58. 7709	56. 6894	57. 92371214
114. 12	C5	48. 2667	48. 4416	49. 1385	48. 37055552
136. 45	C6	73. 6715	74. 1361	73. 8452	73. 76566037
170. 35	C7	5. 4448	5. 3313	5. 4365	5. 424692624
	H19	25. 408	25. 4158	25. 4035	25. 4089517
	H20	23. 7902	23. 7929	23. 7664	23. 7886426
	H21	28. 0682	28. 0783	28. 0638	28. 06955338
	H22	28. 0051	28. 0006	27. 9951	28. 0034824
	H23	28. 0079	28. 005	27. 9969	28. 00647109
	H24	26. 7766	26. 7763	27. 0611	26. 80068313
	H25	26. 8628	26. 7545	26. 9828	26. 8544654
	H26	26. 6134	26. 5057	26. 5328	26. 58815088
	H27	26. 4442	26. 6854	26. 6084	26. 49936336
	H28	29. 7261	29. 7287	29. 7641	29. 72976806
	H29	30. 0901	29. 8824	29. 8018	30. 03013608
	H30	29. 9476	30. 1242	30. 1093	29. 99150765
	H31	30. 9919	30. 4668	31. 5983	30. 95357357
	H32	20. 3265	20. 3297	20. 2986	20. 32468027
					0

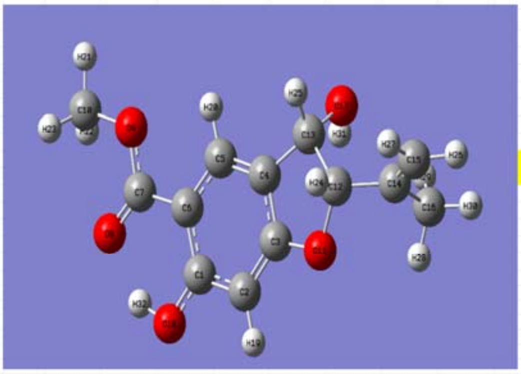

The optimized conformation and dihedral angle (H-2–C-2–C-3–H-3) of compound **3**.

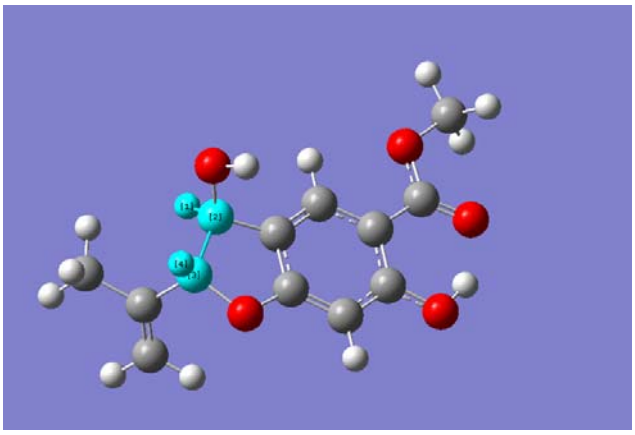

DP4+ analysis of compound **3**.

Functional	Solvent?	Basis Set	Type of Data
mPW1PW91	PCM	6-311+G(d, p)	Shielding Tensors

Nuclei	sp2?	DP4+ Experimental	Isomer 1	Isomer 2	Isomer 3	Isomer 4	Isomer 5	Isomer 6	Is
C	x	156.80	15.0241	9.4137					
C		52.46	133.6925	129.1638					
C		93.49	86.6590	86.6425					
C		76.75	106.9516	105.5670					
C	x	141.28	35.3972	31.1577					
C	x	112.86	70.3386	67.4315					
C		17.48	168.4669	160.8873					
C	x	109.31	86.2897	82.0043					
C	x	152.14	14.8836	10.4863					
C	x	113.09	64.0237	57.9237					
C	x	114.12	52.8292	48.3706					
C	x	136.45	78.9204	73.7657					
C	x	170.35	10.7928	5.4247					

Functional	Solvent?	Basis Set	Type of Data
mPW1PW91	PCM	6-311+G(d, p)	Shielding Tensors

	Isomer 1	Isomer 2	Isomer 3	Isomer 4	Isomer 5	Isomer 6	Isc
sDP4+ (H data)							
sDP4+ (C data)	99.02%	0.98%					
sDP4+ (all data)	99.02%	0.98%					
uDP4+ (H data)							
uDP4+ (C data)	100.00%	0.00%					
uDP4+ (all data)							
DP4+ (H data)							
DP4+ (C data)	100.00%	0.00%					
DP4+ (all data)							
